# A Risk Model Based on Ferroptosis‐Related Genes OSMR, G0S2, IGFBP6, IGHG2, and FMOD Predicts Prognosis in Glioblastoma Multiforme

**DOI:** 10.1111/cns.70161

**Published:** 2025-01-15

**Authors:** Yaqiu Wu, Ling Liu, Zhili Li, Tian Zhang, Qi Wang, Meixiong Cheng

**Affiliations:** ^1^ Department of Neurosurgery Intensive Care Unit, Sichuan Provincial People's Hospital University of Electronic Science and Technology of China Chengdu China; ^2^ Department of Neurosurgery, Sichuan Provincial People's Hospital University of Electronic Science and Technology of China Chengdu China

**Keywords:** Ferroptosis, Gene and Protein Expression, Glioblastoma Multiforme, Multi‐omics, Risk Model

## Abstract

**Background:**

Glioblastoma multiforme (GBM) is a common and highly aggressive brain tumor with a poor prognosis. However, the prognostic value of ferroptosis‐related genes (FRGs) and their classification remains insufficiently studied.

**Objective:**

This study aims to explore the significance of ferroptosis classification and its risk model in GBM using multi‐omics approaches and to evaluate its potential in prognostic assessment.

**Methods:**

Ferroptosis‐related genes (FRGs) were retrieved from databases such as FerrDB. The TCGA‐GBM and CGGA‐GBM datasets were used as training and testing cohorts, respectively. Univariate Cox regression and LASSO regression analyses were performed to establish a risk model comprising five genes (OSMR, G0S2, IGFBP6, IGHG2, FMOD). A Meta‐analysis of integrated TCGA and GTEx data was conducted to examine the differential expression of these genes between GBM and normal tissues. Key gene protein expression differences were analyzed using CPTAC and HPA databases. Single‐cell RNA sequencing (scRNA‐seq) analysis was employed to explore the cell type‐specific distribution of these genes.

**Results:**

The five‐gene risk model demonstrated significant prognostic value in GBM. Meta‐analysis revealed distinct expression patterns of the identified genes between GBM and normal tissues. Protein expression analysis confirmed these differences. scRNA‐seq analysis highlighted the diverse distribution of these genes across different cell types, offering insights into their biological roles.

**Conclusion:**

The ferroptosis‐based risk model provides valuable prognostic insights into GBM and highlights potential therapeutic targets, emphasizing the biological significance of ferroptosis‐related genes in tumor progression.

## Graphical abstract

Molecular mechanism diagram depicting the accurate prognostic evaluation of GBM patients by the prognostic model constructed from OSMR, G0S2, IGFBP6, IGHG2, and FMOD.

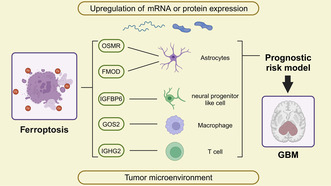

## 1 Introduction

Glioblastoma multiforme (GBM) is the most common and aggressive malignant tumor in the adult central nervous system, with a very poor prognosis. Current treatments such as surgery, radiotherapy, and chemotherapy have limited effectiveness [1, 2, 3, 4, 5]. Therefore, exploring new therapeutic targets, prognostic indicators, and a deeper understanding of its pathogenesis are crucial directions in current GBM research [6]. The development of GBM involves a complex process, including genetic mutations, changes in the tumor microenvironment (TME), and immune cell infiltration. A thorough investigation of these factors can help us gain a comprehensive understanding of the biological characteristics and progression of GBM [7].

In recent years, ferroptosis has garnered attention as a novel form of programmed cell death [8, 9, 10, 11]. Ferroptosis is a cell death mechanism triggered by uncontrolled lipid peroxidation, dependent on the production of iron ions and lipid hydroperoxides [12]. Ferroptosis plays a significant role in tumor pathogenesis, particularly in the initiation, progression, and treatment of tumors [13, 14, 15, 16]. Utilizing the expression profiles of ferroptosis‐related genes (FRGs) can aid in evaluating the prognosis of cancer patients, providing a more precise basis for clinical decision‐making [17]. As the most enriched form of programmed cell death in gliomas, ferroptosis can induce immune suppression and resistance to immune therapy [18]. Thus, inducing ferroptosis has emerged as an attractive strategy for treating gliomas [19].

High‐throughput sequencing and multiomics technologies offer new avenues and perspectives for researching GBM [20]. Through these advanced technologies, we can comprehensively analyze the genetics, transcriptomics, proteomics, and cellular aspects of GBM, identifying new prognostic indicators and potential therapeutic targets [7]. By conducting a systematic analysis of FRGs combined with multiomics data of GBM, we can construct a predictive risk assessment model that provides a more accurate evaluation of patient prognosis [21].

Building on these concepts, this study utilized public GBM sample data from databases, combined with the expression information of FRGs, to develop a ferroptosis‐based GBM prognostic risk assessment model. Linking this model with immune cell infiltration and the TME has revealed the complex biological characteristics and developmental processes of GBM. We believe that this research outcome deepens our understanding of the pathogenesis of GBM, offering new perspectives and evidence for prognostic assessment and personalized treatment of GBM.

## 2 Materials and Methods

### 2.1 Downloading Transcriptome Sequencing Data From Public Databases

The TCGA‐GBM dataset was retrieved from The Cancer Genome Atlas (TCGA) database (https://portal.gdc.cancer.gov/), and RNA expression data (HTSeq‐FPKM) for 168 GBM tumor tissue samples and five normal tissue samples were downloaded. Correspondingly, clinical data for these 168 GBM patients were also gathered. To increase the number of normal control samples, 1152 normal brain tissue samples were searched for and downloaded from the genotype‐tissue expression (GTEx) database (https://www.gtexportal.org/home/index.html).

Furthermore, the “mRNAseq_693” and “mRNAseq_325” data were obtained from the Chinese Glioma Genome Atlas (CGGA) database (http://www.cgga.org.cn/), with a dataset including 247 GBM samples and their clinical data.

A meta‐analysis was conducted on GBM‐related transcriptome RNA sequencing datasets sourced from the Gene Expression Omnibus (GEO) database (https://www.ncbi.nlm.nih.gov/geo/), as shown in Table S1. Additionally, GBM‐related single‐cell RNA sequencing (scRNA‐seq) data GSE140819 were downloaded from the GEO database and extracted brain tissue sample data (GSM4186981) from a single GBM patient. Since these data were from public databases, ethical approval was not required. Refer to Figure S1 for details on the bioinformatic analysis workflow of this study.

### 2.2 FRGs Download

The FRG set was acquired from the FerrDB database (http://www.zhounan.org/ferrdb/current/), and the hsa04216 pathway gene set “Ferroptosis ‐ *Homo sapiens* (human)” was obtained from the KEGG database (https://www.kegg.jp/). Additionally, the ferroptosis gene set “WP_FERROPTOSIS.v2023.1.Hs.gmt” was downloaded from the WikiPathways database (https://www.wikipathways.org/) [22, 23].

### 2.3 Enrichment Analysis of Functional Genes

To better identify the functions of FRGs, enrichment analysis using the R package clusterProfiler was conducted. A total of 54 FRGs were analyzed, and enrichment analysis was performed using Gene Ontology (GO) and the Kyoto Encyclopedia of Genes and Genomes (KEGG). The GO terms were encompassed in biological process (BP), cellular component (CC), and molecular function (MF) categories [24].

### 2.4 Consensus Clustering Analysis

Consensus clustering analysis is an unsupervised clustering method that utilizes a resampling approach to extract a specific number of samples from the given dataset, specifies the number of clusters, *k*, and computes the consistency under different cluster numbers.

This study employed the Consensus Cluster Plus function in R software (v4.3.0) for consensus clustering analysis. Calculating and plotting the consensus cumulative distribution function (CDF) curve assists in determining the optimal *k* value. The Delta Area plot is utilized to calculate the relative change in the area under the CDF curve between *k* and *k*−1, facilitating the selection of the optimal *k* value.

Furthermore, a Cluster‐Consensus Plot was generated to illustrate the Cluster‐Consensus Value of each category under different *k* values. A higher value indicates greater stability, enabling the assessment of the stability differences within the same *k* value and between different *k* values. Principal component analysis was further conducted to validate the clustering performance [25].

### 2.5 Clinical Relevance Analysis of Survival Analysis of Ferroptosis Subtypes

Based on TCGA and CGGA datasets, the R package survival was utilized to examine the correlation between ferroptosis subtypes and overall survival (OS) in GBM patients. Specifically, Kaplan–Meier survival analysis was conducted to compare the OS difference between Cluster A and B groups. Moreover, the OS, disease‐specific survival, and progression‐free interval differences between patients with high and low OSMR expression based on the TCGA‐GBM dataset were compared. Additionally, differences in clinical parameters (age, gender, PRS type, IDH mutation, 1p/19q deletion) between Cluster A and Cluster B groups were evaluated, and the results were illustrated using overlaid bar graphs [26].

### 2.6 TME Analysis

To perform TME analysis, the R package “estimate” was employed. Specifically, calculations of StromaScore, ImmuneScore, and ESTIMATEScore for each sample were conducted. Subsequently, statistical methods were applied to test the differences in scores between Cluster A and Cluster B groups, as well as between high and low‐risk groups [27].

### 2.7 Immune Infiltration Analysis

CIBERSORT, a deconvolution algorithm, was utilized to describe the cellular composition of tissues based on gene expression. This method quantifies the abundance of specific cell types and has been validated by fluorescence‐activated cell sorting (FACS). The CIBERSORT portal contains gene expression data with standard annotations, and the algorithm was run using the LM22 signature and 1000 permutations [28]. This study employed the CIBERSORT algorithm to calculate the relative proportions of 22 immune cells in each sample through 1000 simulations. The results indicated differences in immune cell content between Cluster A and Cluster B groups (*p* < 0.05).

### 2.8 Gene Set Variation Analysis (GSVA)

GSVA is a nonparametric unsupervised analysis method that transforms the gene expression matrix between different samples into a matrix of gene set expression between samples to assess the enrichment of various signaling pathways across different samples [29]. In this study, the R package GSVA was utilized with the data from “c5.go.symbols.gmt” and “c2.cp.kegg.symbols.gmt” to analyze the association between ferroptosis subtypes, prognosis risk model, and GO and KEGG pathways.

Furthermore, the single‐sample gene set enrichment analysis (ssGSEA) algorithm provided by the R package GSVA was employed. This algorithm, using the immune cell markers proposed by Bindea et al. in their Immunity article [30], calculated the immune infiltration status of each GBM sample. The correlation between 22 immune cells and the expression of OSMR, G0S2, IGFBP6, IGHG2, and FMOD was investigated.

### 2.9 Correlation Analysis With Immune Checkpoints

A total of 47 common immune checkpoints, including IDO1, LAG3, CTLA4, TNFRSF9, ICOS, CD80, PDCD1LG2, TIGIT, CD70, TNFSF9, ICOSLG, KIR3DL1, CD86, PDCD1, LAIR1, TNFRSF8, TNFSF15, TNFRSF14, IDO_2_, CD276, CD40, TNFRSF4, TNFSF14, HHLA2, CD244, CD274, HAVCR2, CD27, BTLA, LGALS9, TMIGD2, CD28, CD48, TNFRSF25, CD40LG, ADORA2A, VTCN1, CD160, CD44, TNFSF18, TNFRSF18, BTNL2, C10orf54, CD200R1, TNFSF4, CD200, and NRP1 were identified. Using the R package “corrplot,” correlation analysis between the expression of OSMR, G0S2, IGFBP6, IGHG2, FMOD, and these 47 common immune checkpoints based on the TCGA‐GBM dataset was conducted [31].

### 2.10 Differential Gene Expression Analysis

Based on the TCGA‐GBM dataset, the R package “limma” was employed to identify genes with differential expression between cluster A and cluster B groups using a threshold of |logFC| > 1 and *p* < 0.05 [32].

### 2.11 Univariate cox Analysis and LASSO Regression Analysis

Utilizing survival information from GBM patients in the TCGA database, univariate Cox analysis using the R package “Survival” was conducted to select key module genes. The identified genes were visually represented using the R package “Forestplot” to observe their survival risk. A hazard ratio (HR) > 1 was set as the criterion for high‐risk genes, and a statistically significant result was defined as Log‐rank *p* < 0.05.

Subsequently, LASSO regression analysis was performed using the R package “Glmnet” to determine the expression levels of each gene. Samples with a risk score lower than the average RS of all samples were classified as low‐risk, while those above were deemed high‐risk. The optimal number of candidate genes with the minimum error and their corresponding log(λ) values were identified to further refine the model [33].

### 2.12 Construction of Prognostic Risk Model

Using the TCGA‐GBM dataset as the training set and the CGGA‐GBM dataset as the testing set, univariate Cox analysis was conducted on the key module genes using the R package “survival” to identify prognostically relevant genes. Genes with a *p*‐value < 0.05 were selected and subjected to multivariate Cox analysis to establish the prognostic risk model. The RS for each patient was calculated as the sum of the regression coefficients of genes and their expression levels.

Patients were stratified into high‐risk and low‐risk groups based on the median RS, and Kaplan–Meier survival analysis was used to compare the OS differences between the two groups with a significance level of *p* < 0.05. Additionally, ROC analysis was conducted using the R packages “timeROC” and “pROC” to assess the accuracy of the prognostic risk model through the evaluation of ROC curves and the area under the ROC curve (AUC value). These metrics were used to determine the sensitivity and specificity of the survival analysis model [34].

### 2.13 Univariate and Multivariate Independent Prognostic Analysis

Similarly, utilizing the TCGA‐GBM dataset as the training set and the CGGA‐GBM dataset as the testing set, we predicted the RS of samples through LASSO regression and conducted univariate independent prognostic analysis using the R package “Survival” to initially assess the relationship between clinical attributes and prognostic survival. Furthermore, multivariate independent prognostic analysis was performed to accurately evaluate the impact of clinical attributes on prognostic survival outcomes. Forest plots were generated using the R package “Forestplot” to observe the survival risk of clinical attributes, with HR > 1 serving as the selection criterion for attributes and a Log‐rank *p*‐value < 0.05 considered statistically significant [35].

### 2.14 Meta‐Analysis

When conducting meta‐analysis using the R package “Meta,” methods for evaluating combined effects typically involved mean difference (MD) and 95% confidence interval (CI).

To assess the combinability of individual studies, tests for heterogeneity (i.e., tests for the homogeneity of statistical quantities) needed to be performed. This often involved the *Q* test, chi‐square test, and assessing *I* ^2^ values and *p*‐values to evaluate the presence and magnitude of heterogeneity. If the results indicated no statistically significant heterogeneity among the studies (*p* > 0.05, *I* ^2^ < 50%), the studies were found to be combinable, with the differences arising from sampling errors. A fixed‐effect model was used for the combined analysis in such cases. Conversely, if significant statistical heterogeneity exists among the studies (*p* < 0.05, *I* ^2^ > 50%), a random‐effects model was employed for the combined analysis. Additionally, sensitivity analysis was performed using the leave‐one‐out method to evaluate the stability of the analytical results [36].

### 2.15 Protein Expression Data Analysis

Data from the Clinical Proteome Tumor Analysis Consortium (CPTAC) and the Human Protein Atlas (HPA) (https://www.proteinatlas.org/) databases were utilized to investigate the protein expression differences of OSMR, G0S2, IGFBP6, IGHG2, and FMOD in tumor tissue samples from GBM patients compared to normal control tissue samples. The aim was to further explore these differences [37].

### 2.16 scRNA‐Seq Data Analysis

After obtaining the expression matrix using the official software Cellranger from 10 × Genomics, initial cell filtering was performed by selecting cells expressing a gene count (nFeature) > 200 and < 5000 (nFeature_RNA), while also excluding cells with a mitochondrial gene percentage (percent.mt) exceeding 10%.

Subsequently, data quality postfiltering was inferred by combining unique molecular identifiers (UMI) and gene correlation analysis. Principal component analysis was carried out using the R package Seurat [38] to reduce the dimensionality of the high‐variable gene expression matrix. JackStrawPlot and ElbowPlot functions were used to select the principal components necessary for downstream analysis. Clustering algorithms based on graphs were utilized, along with UMAP for two‐dimensional visualization. Finally, cells were annotated based on known marker genes [39]. All analyses were conducted using R (v4.3.0) software.

### 2.17 Cell Culture

Normal human astrocytes, HA (GN‐H120, Shanghai Genomeditech Co. Ltd.), primary mouse astrocytes MA (CM‐M081, Shanghai Genomeditech Co. Ltd.), and A172 cells (CBP60300) were purchased from Shanghai Genomeditech Co. Ltd. Human glioblastoma cell lines LN229 (CBP60302), BT325 (CBP61012), U251 (CBP60300) were acquired from Nanjing KeyGen Biotech Co. Ltd. GL261 cells were cultured in RPMI‐1640 medium (R8758, Gibco, USA) supplemented with 10% fetal bovine serum (FBS, 10091148, Gibco), 100 μg/mL streptomycin, and 100 U/mL penicillin (15140148, Gibco). HMC3 and A172 cells were cultured in MEM medium (41090101, Gibco) with 10% FBS, 1% nonessential amino acids (NEAA, 11140076, Gibco), and 1 mM sodium pyruvate (NaP, 11360070, Gibco). LN229 cells were cultured in DMEM medium (11330057, Gibco) with 10% FBS, 100 μg/mL streptomycin, and 100 U/mL penicillin. Mouse glioma cells GL261 (BNCC341792, Beina Biotech, Henan Industrial Microbial Strains Engineering Research Center) were cultured in GL261 complete medium (BNCC360453, Beina Biotech, Henan Industrial Microbial Strains Engineering Research Center). Human spleen T cells (Mingzhou, 4229, Zhejiang, China) and human CD8^+^ T cells were cultured in CTS OpTmizer T Cell Expansion SFM medium (Thermo Fisher, A1048501, USA). CD8^+^ T cells were isolated using Dynabeads CD8 Positive Isolation Kit (Thermo Fisher, 11333D, USA) from T cells. All cell lines were cultured in a 37°C humidified incubator with 5% CO_2_, and the medium was changed 3–4 times per week based on cell growth. Passages were performed when cell confluence reached approximately 80% [40, 41].

### 2.18 Co‐Culture of Glioblastoma Cells and Macrophages

Glioblastoma cells and THP‐1 cells were mixed at a 1:1 ratio and added to a 12‐well cell culture plate. After co‐culturing for 24 h, the polarized macrophages were collected using a cell scraper. These cells were identified using flow cytometry with fluorescently labeled antibodies, including anti‐CD80 (12‐0801‐82, Thermo Fisher), anti‐CD86 (11‐0862‐82, Thermo Fisher), anti‐CD163 (17‐1639‐42, Thermo Fisher), and anti‐CD206 (25‐2061‐82, Thermo Fisher).

### 2.19 shRNA‐Mediated Gene Knockdown, Lentiviral Infection, and Construction of Overexpression Cell Lines

The OSMR gene was knocked down via shRNA interference. Plasmids containing a single luciferase reporter gene were co‐transfected with helper plasmids into 293T cells. After validation, amplification, and purification, lentiviral packaging was obtained. Lentiviral infection was carried out in GL261, GL261‐luc, or LN229 cells, with OSMR knockdown (OSMR‐KD) or control cell lines (OSMR‐NC). For GL261, the control cell line was the mouse astrocyte cell line (MA), and for LN229, the control was the human astrocyte cell line (HA). Cells (5 × 10^5^) were seeded in six‐well plates, and when cell confluence reached 70%–90%, a medium containing an appropriate amount of packaged lentivirus (MOI = 10, working titer approximately 5 × 10^6^ TU/mL) and 5 μg/mL polybrene (TR‐1003, Merck, USA) was added for transfection. After 4 h, an equal volume of medium was added to dilute polybrene. The medium was replaced after 24 h, and 48 h post‐transfection, transfection efficiency was observed via luciferase reporter genes. Stable transduced cell lines were selected using 60 μg/mL ampicillin (A100339, Sangon Biotech, Shanghai, China). The infection efficiency was determined via RT‐qPCR and Western blot, and the most efficient lines were selected for further experiments. Each experiment was repeated three times. shRNA sequences are listed in Table S2 [42, 43, 44, 45].

Overexpression of OSMR was achieved by transfecting GL261 glioma cells with OSMR‐expressing plasmids, creating an OSMR overexpression cell line (oe‐OSMR). Cells without overexpression were used as the control (wt‐OSMR). After 48 h of transfection, protein and RNA levels were analyzed to verify the knockdown and overexpression efficiency [46].

### 2.20 RT‐qPCR

Cells were lysed using the Trizol reagent (10296010, Invitrogen, Thermo Fisher, USA), and total RNA was extracted. The quality and concentration of RNA were measured using a UV–visible spectrophotometer (ND‐1000, Nanodrop, Thermo Fisher, USA). Reverse transcription was performed using the PrimeScript RT‐qPCR kit (RR086A, TaKaRa, Mountain View, CA, USA). Real‐time quantitative reverse transcription polymerase chain reaction (RT‐qPCR) was carried out on a LightCycler 480 system (Roche Diagnostics, Pleasanton, CA, USA) using SYBR Premix Ex TaqTM (DRR820A, TaKaRa). GAPDH was used as the reference gene. Primers for amplification were designed and provided by Shanghai General Biosystems Co. Ltd. Table S3 for primer sequences. The expression of the target gene in the experimental group relative to the control group was calculated using the $2^{-∆∆C_{t}}$ method, where ΔΔCT = Δ*C*_t_ experimental group—Δ*C*_t_ control group, and Δ*C*_t_ = target gene *C*_t_—reference gene *C*_t_ [47, 48].

### 2.21 CCK8 Assay

Log‐phase cells from each group were counted and seeded into 96‐well plates at a density of 2 × 10^3^ cells per well (100 μL) and preincubated for 24 h. For each cell group, five parallel control wells were set up, with cell‐free medium wells serving as blanks, totaling four plates to be seeded. The plates were then cultured under standard conditions of 5% CO_2_ at 37°C. Every 24 h, one plate was removed for measurement. Ten microliters of CCK8 (K1018, Apexbio, USA) was added to each well, followed by a 2‐h incubation at 37°C. The optical density (OD) values at 450 nm in the wells were measured using an enzyme immunoassay at four‐time points (0, 24, 48, and 72 h) [49].

### 2.22 Co‐Culture of Tumor Cells and CD8 ^+^T Cells

Tumor and CD8^+^T cells were co‐cultured using Transwell inserts with a pore size of 0.4 μm (3422, Corning). CD8^+^T cells were seeded in the upper chamber of the Transwell (10^5^ cells/well), while tumor cells were seeded in the lower chamber of the Transwell (5 × 10^4^ cells/well). CD8^+^T cells were harvested for subsequent analysis after 48 h. The cytotoxicity of CD8^+^ T cells was evaluated using FITC‐Mouse‐anti‐Human‐Granzyme B (GzmB) (20 μL/test, BD Bioscience, 560211, USA) or Alexa Fluor 488‐Mouse‐anti‐Human‐Ki67 (5 μL/test, BD Bioscience, 561165, USA) and APC‐Mouse‐anti‐Human‐CD8 (5 μL/test, BD Bioscience, 340584, USA). Cell apoptosis rate was detected using flow cytometry [50], and the data obtained were analyzed using BD FACSDiva software.

### 2.23 Transwell Assay

To assess cell migration ability, log‐phase cells from each group were resuspended in serum‐free DMEM medium and adjusted to a cell density of 5 × 10^5^ cells per milliliter. Subsequently, 100 μL of cell suspension was seeded in the upper chamber of the Transwell, and each well was filled with 700 μL of DMEM medium containing 15% FBS in a 24‐well plate. After seeding the cells in the upper chamber of the Transwell, they were incubated in a cell culture incubator for 24 h. Following incubation, the medium in the upper chamber was discarded, and the cells were fixed with 95% ethanol after two PBS washes. Staining was performed with 0.5% crystal violet, and any remaining cells on the upper chamber surface were wiped off with a cotton swab. The cells on the lower chamber surface were observed under a microscope to count the migrated cells. For invasion assessment, 100 μL of serum‐free DMEM mixed in a 6:1 dilution with Matrigel (354230, Corning) was added to the upper chamber surface of the Transwell membrane. The remaining procedures were similar to those for cell migration assessment [49].

### 2.24 Detection of Ferrous Iron Ions

The iron content detection kit (MAK025‐1KT, Sigma‐Aldrich, USA) was utilized to assess the enrichment of Fe^2+^ in cell and tissue suspensions from various groups. After digestion with 0.25% trypsin and centrifugation, samples were treated with a fourfold iron determination buffer. The supernatant obtained after low‐temperature centrifugation was collected as the test solution. Subsequently, 50 μL of the test sample was added to a 96‐well cell culture plate, with 5 μL of iron ion analysis buffer added to each iron ion standard sample well. Incubation was conducted at room temperature for 30 min, followed by adding 100 μL of iron ion probe reagent to each sample well and iron ion standard sample well. The plates were then incubated at room temperature for 60 min under light avoidance to ensure adequate probe loading. For the analysis of ferrous iron ions, 50 μL of the sample was added to the sample wells of the 96‐well plate, along with 5 μL of the analysis buffer in each well. Incubation of iron standard samples and test samples was carried out at 25°C for 30 min. Subsequently, 100 μL of iron ion probe was added to each well containing iron standard and test samples, mixed thoroughly, and left for 60 min at 25°C under light avoidance. The optical density values at 593 nm were measured using a microplate reader [51].

### 2.25 Detection of Reactive Oxygen Species (ROS)

The ROS detection kit (Beyotime, S0033S, Shanghai) was utilized for ROS detection following the manufacturer's instructions. Initially, the reagent was added to the sample for reaction as per the kit instructions, and then the fluorescence images of the samples were captured using a fluorescence microscope. The collected images were quantitatively analyzed using ImageJ software or similar image analysis software to calculate the average fluorescence intensity of six randomly selected areas in each experiment. Finally, the results were expressed as multiples of the fluorescence intensity of the control group [52], providing an evaluation of ROS production in the cells.

### 2.26 ELISA

Cell lysates from each group and mouse cerebrospinal fluid were subjected to experiments following the instructions of the ELISA kits to detect the levels of the ferroptosis key factors GSH and GPX4 in cells, as well as the levels of IFN‐γ, TNF‐α, IL‐10, and IL‐13 in mouse cerebrospinal fluid. The following kits were used: GSH (JN20809, Jining Industrial, Shanghai), GPX4 (JL46163, Future Industries, Shanghai), IFN‐γ (JL10967, Future Industries, Shanghai), TNF‐α (JN17113, Jining Industrial, Shanghai), IL‐10 (JL20242, Future Industries, Shanghai), and IL‐13 (JL20247, Future Industries, Shanghai) [53].

### 2.27 Western Blot

Cell proteins were extracted using the tissue protein rapid extraction kit (EX2410) and cell protein extraction kit (EX2170) from Solbio Company. The protein concentration was determined using the BCA protein assay kit (Sigma, BCA1‐1KT). SDS‐PAGE electrophoresis with 10%–12% gel was performed with 20 μg of equal protein loaded per lane, then transferred to polyvinylidene difluoride membrane (EMD Millipore, Billerica, MA). The membrane was then blocked with 5% BSA for 2 h, washed with PBS, and incubated overnight at 4°C with primary antibodies (OSMR (ab282577, 1:1000, Abcam); GAPDH (ab8245, 1:2000, Abcam); ACSL4 Rabbit mAb (A20414, 1:1000, Ab clone); Anti‐5 Lipoxygenase/5‐LO antibody (ab53514, 1:2000, Abcam); Anti‐beta Actin antibody (ab8227, 1:1000, Abcam)). After washing, the membrane was incubated with goat anti‐rabbit horseradish peroxidase secondary antibody (Abcam, ab6721, 1:2000, UK) for 2 h at room temperature. Finally, chemiluminescence was performed using the iBright FL1500 imaging system (Thermo Fisher), and the protein bands were scanned for grayscale using AlphaView SA software (Version: 3.4.0) [54]. The relative protein expression level was calculated by comparing the relative grayscale values of the target bands to the internal control GAPDH, and the experiment was repeated three times.

### 2.28 Flow Cytometry for Cell Death Detection

Flow cytometry is utilized to assess cell death rates. Essentially, tumor cells (1 × 10^5^/well) were collected, washed with chilled PBS, and then stained in the dark for 15 min using the detection kit (APOAF‐20TST, Sigma‐Aldrich, USA). Subsequently, the residue was suspended in 400 μL binding buffer, followed by adding 5 μL Annexin‐V staining agent provided in the kit. Cell analysis was performed using a flow cytometer [55]. Cells in the upper‐right quadrant displaying the phenotype Annexin V^+^PI^+^ represent late apoptotic cells; cells in the lower‐right quadrant displaying Annexin V^+^PI^−^ represent early apoptotic cells; cells in the upper‐left quadrant displaying Annexin V^−^PI^+^ are indicative of necrotic cells, and cells in the lower‐left quadrant displaying Annexin V^−^PI^−^ represent viable cells [56]. Moreover, cells infected with OSMR‐KD were treated with 1 μM Fer‐1, 20 μM pan‐caspase inhibitor Z‐VAD‐FMK, or 10 μM RIPK3 inhibitor GSK872 for 30 h. The cells were then stained with SYTOX Green (Invitrogen) and subjected to flow cytometry to analyze cell death indicators. For 5‐ethynyl‐2′‐deoxyuridine (EdU) staining, cells were seeded onto circular coverslips in a 24‐well plate for 24 h, followed by staining with Yefluor 594 EdU Imaging Kit (40276ES60, YEASEN) according to the manufacturer's instructions [57].

Flow cytometry was used to analyze macrophage expression in mouse glioma tissues from the OSMR‐NC and OSMR‐KD groups. First, collected peripheral blood was digested in RPMI 1640 medium (11875119, Gibco) with 10 U/mL collagenase I (17018029, Gibco) and 30 U/mL DNase I (18047019, Invitrogen) at 37°C for 60 min. The cell suspension was filtered through a 40 μm nylon filter. After lysing the red blood cells, the cells were washed twice with complete RPMI 1640 medium and stained at 4°C with the following specific antibodies: anti‐CD80‐APC (560016), anti‐CD86‐PE (553692), anti‐CD163‐BV605 (569202), and anti‐CD206‐PE (568273), all purchased from BD Biosciences. Finally, the cells from each group were washed twice with PBS and sorted using a flow cytometer (Beckman Coulter, USA). Data were analyzed using FlowJo software [58].

### 2.29 Cell Sorting via Flow Cytometry

Cell samples were washed with PBS, resuspended, and prepared for further use. The excised mouse tumors were digested in RPMI medium containing 20 μg/mL DNase I (11284932001, Merck KGaA, Darmstadt, Germany) and 5 μg/mL Collagenase IV (C4‐BIOC, Merck KGaA, Darmstadt, Germany) for 1 h at 37°C, followed by digestion for an additional hour. The resulting mixture was filtered using a 70 μm Corning Cell Strainer (CLS431751, Merck KGaA, Darmstadt). The single‐cell suspension was treated with red blood cell lysis buffer (00‐4333‐57, Invitrogen), washed, and resuspended in PBS. After removing dead cells with Percoll (P1644, Merck KGaA, Darmstadt, Germany) per the manufacturer's instructions, cells were resuspended in 100 μL of PBS at a concentration of 1 × 10^7^ cells/mL. When intracellular antigens needed detection, cells were pretreated with 0.5% Tween 20 (P2287, Merck KGaA, Darmstadt, Germany) for 5 min before incubating with primary antibodies. The samples were then incubated with antibodies at 4°C and analyzed using a flow cytometer (BD LSRFortessa, BD Bioscience, USA). The specific antibodies used were: FITC anti‐CD45 (103108, BioLegend, Canada), PE/Cy5 Anti‐CD3 (ab25531, Abcam, Cambridge, UK), PerCP‐Cyanine5.5 anti‐CD8 (45‐0081‐82, Invitrogen, Thermo Fisher Scientific Inc., Shanghai, China), PE anti‐CD4 (12‐0041‐82, Invitrogen, Thermo Fisher Scientific Inc., Shanghai, China), PE anti‐Ki67 (12‐5698‐82, Invitrogen, Thermo Fisher Scientific Inc., Shanghai, China), eFluor 450‐Granzyme B (GzmB) (50‐8898‐82, Invitrogen, Thermo Fisher Scientific Inc., Shanghai, China), and the data were analyzed using BD FACSDiva software [59].

### 2.30 Mouse Model Establishment and Treatment

Male BALB/c nude mice aged 4 weeks were obtained from Beijing Vital River Laboratory Animal Technology Co. Ltd. (Beijing, China). All animal research and experimental procedures were approved by our institution's animal ethics committee and complied with the Guide for the Care and Use of Laboratory Animals. Female C57BL/6J wild‐type mice were procured from Beijing Vital River Laboratory Animal Technology Co. Ltd. T cell and B cell‐deficient NOD‐SCID mice from Jackson Laboratory. All mice were housed in a specific pathogen‐free environment at our institution's animal facility, with a temperature of 20°C–25°C and humidity maintained between 60% and 65%. The mice had free access to food and water in a 12‐h dark/light cycle to acclimatize before experimentation [60]. In vivo, animal experiments were approved by the Animal Care and Use Committee of Nantong Tumor Hospital and complied with animal welfare legislation in China. The animal studies followed the ARRIVE guidelines.

The GL261 luciferase‐expressing cells were transfected with lentiviral vectors carrying either OSMR knockdown (OSMR‐KD) and its negative control, or OSMR overexpression (OE‐OSMR) and its control (WT‐OSMR). The mice were randomly divided into different experimental groups. On day 0, approximately 5 × 10^5^ cells in 5 μL were injected into the right brains of the mice using a stereotactic device. From day 7 onward, bioluminescence imaging of intracranial heterotopic transplants was performed weekly. Each experimental group consisted of five mice, except for the survival experiments, which included 10 mice per group. Survival time was recorded, and the brains of the mice were collected for further research [61, 62].

### 2.31 Immunohistochemistry (IHC) Staining

Tumor tissue samples were obtained and prepared into paraffin sections. The sections were dewaxed and gradually dehydrated with alcohol. Tissue retrieval was carried out using antigen repair solution and cooling with distilled water. Normal goat serum blocking solution (provided by Hai Haoran Biotechnology Co. Ltd., China) was applied to the tissue sections and left at room temperature for 20 min. The excess liquid on the slides was then removed. OSMR antibody (10982‐1‐AP, 1:100, Proteintech), CD3 (ab16669, 1:100, Abcam), CD4 (ab133616, 1:500, Abcam), CD8 (ab217344, 1:2000, Abcam), and Ki67 antibody (ab15580, 1:100, Abcam) were added to the tissue sections and incubated overnight at 4°C. Subsequently, the sections were washed thrice in 0.1 M PBS for 5 min each. Goat anti‐rabbit IgG secondary antibody (ab6721, 1:1000, Abcam, UK) was then added to the tissue sections and incubated at 37°C for 20 min. This was followed by the addition of horseradish peroxidase‐conjugated streptavidin protein working solution (0343‐10000 U, Easybio Technology, China) and incubation at 37°C for 20 min, followed by color development using DAB (ST033, Guangzhou Weijia Technology Co. Ltd., China). After color development, the slides were rinsed with water and counterstained with hematoxylin for 1 min. Finally, counterstaining was neutralized using 1% ammonia water, dehydrated with alcohol, and coverslipped with neutral resin [63]. The stained slides were observed, photographed, and saved under an optical microscope (CX43, Olympus, Japan). The signal intensity of the IHC staining was quantified using ImageJ software (1.48u, National Institutes of Health), with the experiment repeated three times [64].

### 2.32 Ki‐67 Staining

70% ethanol was prepared and cooled to −20°C. The target cells were prepared and washed twice with 1× PBS. They were centrifuged at 350 *g* for 5 min, and the supernatant was discarded. The cell pellet was vortexed, and while being vortexed, 3 mL of prechilled 70% ethanol was gradually added dropwise to the cell suspension. Vortexing was continued for 30 s, followed by incubation at −20°C for 1 h. The cells were washed three times with cell staining buffer and resuspended in cell staining buffer at a concentration of 0.5–10 × 10^6^ cells/mL. An appropriate amount of Ki‐67 antibody for flow cytometry (#9449, Cell Signaling) was added to 100 μL of cell suspension and incubated in the dark at room temperature for 30 min. The cells were then washed twice with cell staining buffer and resuspended in 0.5 mL of cell staining buffer for flow cytometric analysis.

### 2.33 Statistical Analysis

All data were derived from three independent experiments and presented as mean ± standard deviation (Mean ± SD). The Shapiro–Wilk tests were used to assess the normality of the data. For data that did not follow a normal distribution, nonparametric tests were employed.

For comparisons between two groups, if the data were normally distributed, an unpaired *t*‐test was used; if not, the Mann–Whitney *U* test was applied. For comparisons among three or more groups, one‐way analysis of variance (ANOVA) was used for normally distributed data, with Tukey's post hoc test for multiple comparisons if significant differences were found. For nonnormally distributed data, the Kruskal–Wallis test was used, followed by Dunn's post hoc test for multiple comparisons. For comparisons of data across different time points, two‐way ANOVA was used for normally distributed data with the Bonferroni post hoc test, while the Friedman test was applied for nonnormally distributed data, followed by appropriate nonparametric post hoc tests.

All statistical analyses were performed using Prism 8.0 software, and a *p*‐value of < 0.05 was considered statistically significant.

## 3 Results

### 3.1 Prognostic and Immune Infiltration Analysis Based on FRGs

Numerous studies indicate the significant role of ferroptosis in tumor progression. Ferroptosis‐related biomarkers are gradually finding applications in clinical diagnosis and prognosis assessment and showing great potential [65, 66, 67]. Therefore, this research focuses on ferroptosis subtyping and reveals the prognostic value of ferroptosis subtyping and the risk model constructed in GBM patients through multiomics technologies.

Initially, we obtained 101 genes by downloading FRGs set from the FerrDB database with a threshold of Score ≥ 2. Additionally, we acquired 41 genes from the “Ferroptosis ‐ *Homo sapiens* (human)” pathway gene set from the KEGG database and 64 genes from the “WP_FERROPTOSIS.v2023.1.Hs.gmt” gene set from the WikiPathways database. By taking the intersection of these three gene sets and pairwise intersections, we identified 54 genes (Figure S2A). Subsequently, through GO and KEGG enrichment analysis, we further confirmed that these 54 genes are crucial FRGs (Figure S2B,C).

**FIGURE 1:**
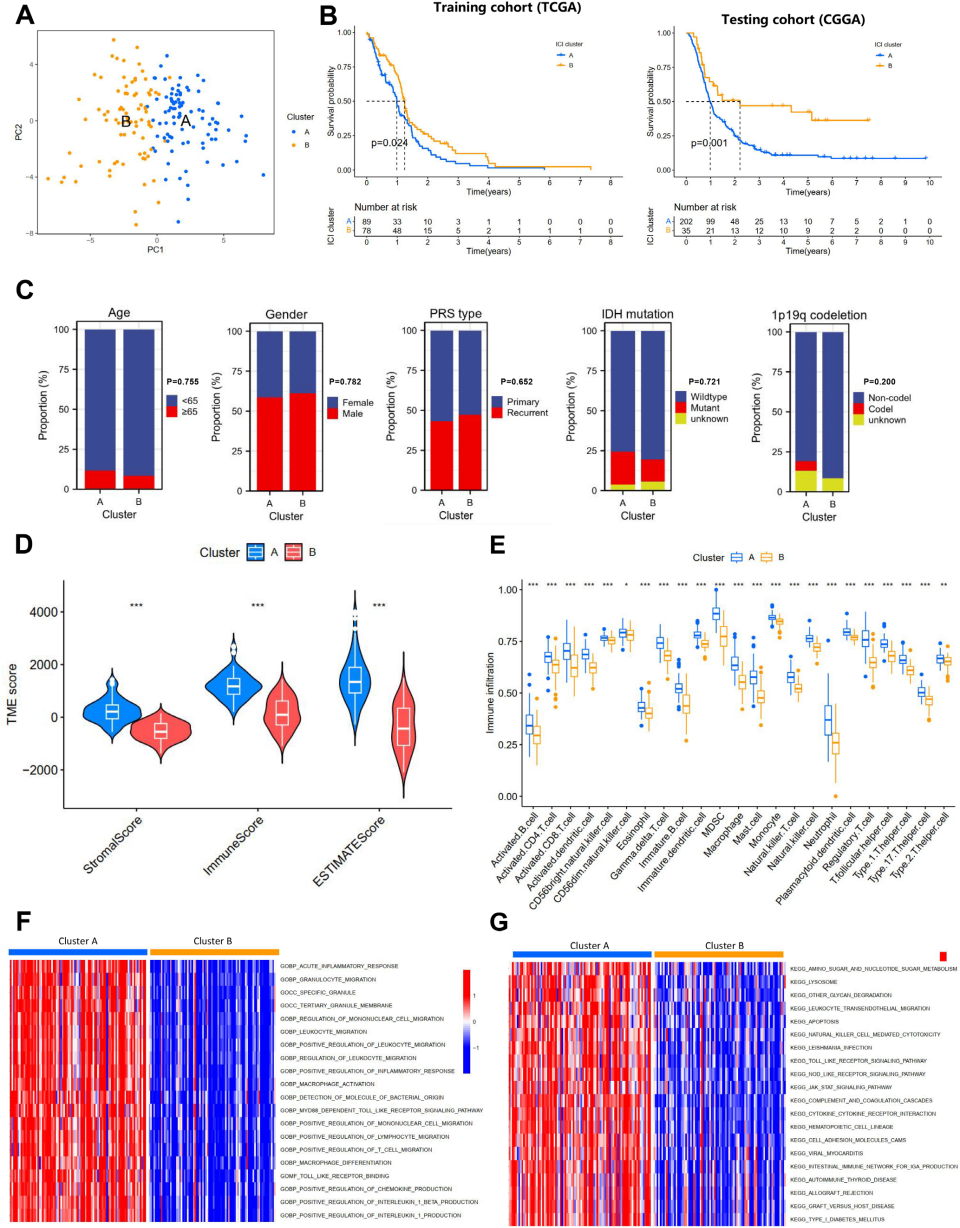
Prognostic analysis of consensus clustering and ferroptosis subtypes. (A) Principal component analysis results of samples from cluster A and cluster B groups; (B) Survival curves of patients in cluster A and cluster B groups in the training cohort (TCGA) and testing cohort (CGGA); (C) Comparison of clinical parameters between patients in cluster A and cluster B groups, including age, gender, PRS type, IDH mutation, 1p/19q codeletion; (D) Stromal score, immune score, and ESTIMATE score of samples in cluster A and cluster B groups; (E) Differential abundance of 22 immune cells between cluster A and cluster B groups; (F) GSVA‐GO(BP) analysis results for cluster A and cluster B groups; (G) GSVA‐KEGG analysis results for cluster A and cluster B groups. *p < 0.05, **p < 0.01, ***p < 0.001.

Next, we performed a consistent clustering analysis of GBM patient tumor tissue samples based on the 54 FRGs. Using the TCGA‐GBM dataset as the training cohort and the CGGA‐GBM dataset as the testing cohort, the clustering results indicated that at *k* = 2, the clustering matrix was more “clean,” indicating a better subtyping effect (Figure S3A–D). The CDF curve was flattest at *k* = 2, with a sharp decrease in coefficients after *k* = 2 (Figure S3B,C). Principal component analysis showed better dispersion when samples were classified into cluster A and cluster B (Figure 1A). Thus, we divided the tumor tissue samples of GBM patients into two clusters for further survival and clinical correlation analysis.

Survival analysis revealed that in both the training and testing cohorts, patients in cluster B had significantly better survival rates compared to those in cluster A (Figure 1B). Furthermore, cluster B patients had lower rates of being ≥ 65 years old, IDH mutation, and 1p/19q codeletion, and higher rates of being male and recurrent compared to cluster A (Figure 1C). This suggests that ferroptosis subtyping holds good prognostic value in GBM.

Using the ESTIMATE algorithm, we calculated the stromal and immune scores for each sample, showing that in cluster B, the StromalScore, ImmuneScore, and ESTIMATEScore were lower compared to cluster A (Figure 1D). Utilizing the CIBERSORT algorithm to display differences in immune cells between the two clusters, we found significant variations in all immune cells between clusters A and B, with lower immune cell content in cluster B than in cluster A (Figure 1E). Additionally, based on “c5.go.symbols.gmt” and “c2.cp.kegg.symbols.gmt,” we conducted GSVA, revealing significant differences in BP and KEGG pathways between the two clusters (Figure 1F,G). These results indicate a close association between ferroptosis subtyping and the TME and immune infiltration in GBM.

### 3.2 The Risk Model Constructed by OSMR, G0S2, IGFBP6, IGHG2, and FMOD Has Good Prognostic Value in GBM Patients and Is Closely Related to Immune Infiltration

**FIGURE 2:**
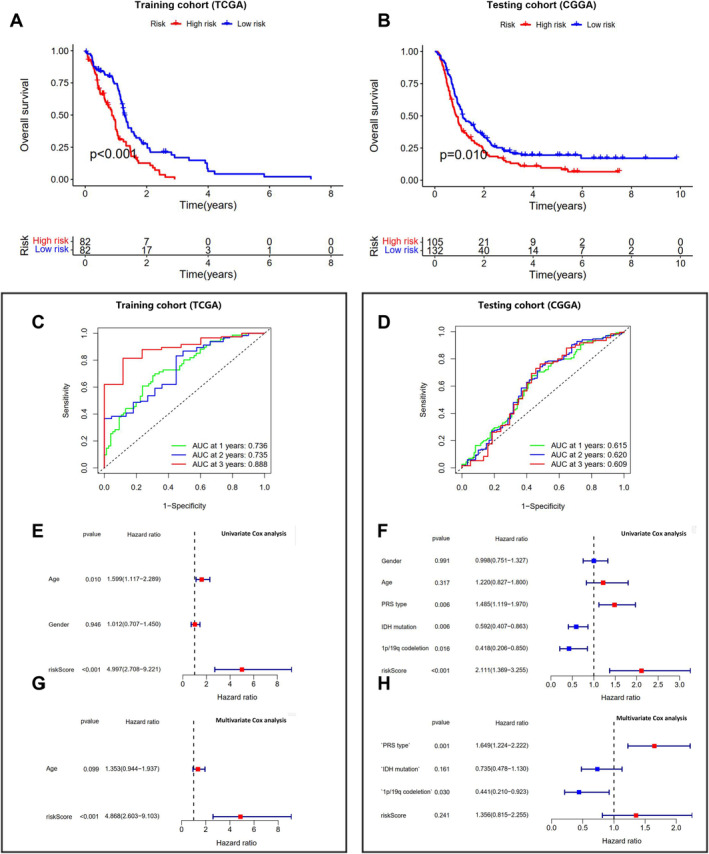
Prognostic analysis of risk model constructed from differential genes based on ferroptosis subtypes. (A) Kaplan–Meier survival curve analysis of high‐risk and low‐risk patients in the training cohort; (B) Kaplan–Meier survival curve analysis of high‐risk and low‐risk patients in the testing cohort; (C, D) ROC curves predicting 1, 2, and 3‐year survival rates of GBM patients in the training and testing cohorts based on the risk model; (E, F) univariate COX regression forest plot analysis in the training and testing cohorts; (G, H) Multivariable COX regression forest plot analysis in the training and testing cohorts. HR represents the risk rate, where an HR > 1 signifies high risk and < 1 signifies low risk. The distribution of HRs to the right indicates high risk, while to the left indicates low risk.

Further analysis of the differences in gene expression between cluster A and cluster B in GBM patients using the TCGA‐GBM dataset, with a threshold of |logFC| > 1 and *p* < 0.05, identified 342 differentially expressed genes (Figure S4A). Single‐factor COX analysis identified 101 genes associated with the prognosis of GBM patients. Subsequently, a risk model composed of five genes (OSMR, G0S2, IGFBP6, IGHG2, and FMOD) was successfully constructed using LASSO regression analysis (Figure S4B,C). As shown in (Figure 2A), patients in the high‐risk group in the training cohort had significantly lower survival rates than those in the low‐risk group, a result validated in the testing cohort (Figure 2B). The RS for each patient was calculated using the formula: RS = (0.37Exp OSMR) + (0.18Exp G0S2) + (0.23Exp IGFBP6) + (0.03Exp IGHG2) + (0.08*Exp FMOD). Moreover, the separation of samples between the high and low‐risk groups was evident, with a higher number of deceased patients and shorter survival times in the high‐risk group, consistent in both the training and testing cohorts (Figure S4D–I).

ROC analysis demonstrated that the risk model accurately predicted the 1‐, 2‐, and 3‐year survival rates of GBM patients in the training cohort (Figure 2C), with slightly inferior predictive ability in the testing cohort (Figure 2D). Single‐factor COX analysis showed that in both cohorts, the risk model accurately predicted the prognosis of GBM patients (Figure 2E,F). Furthermore, multifactor COX analysis revealed that in the training cohort, the risk model accurately predicted patient prognosis, predominately in terms of Age, although not significantly different in the testing cohort (Figure 2G,H). These findings suggest that the risk model based on OSMR, G0S2, IGFBP6, IGHG2, and FMOD was successfully constructed and holds good prognostic value in GBM patients.

**FIGURE 3:**
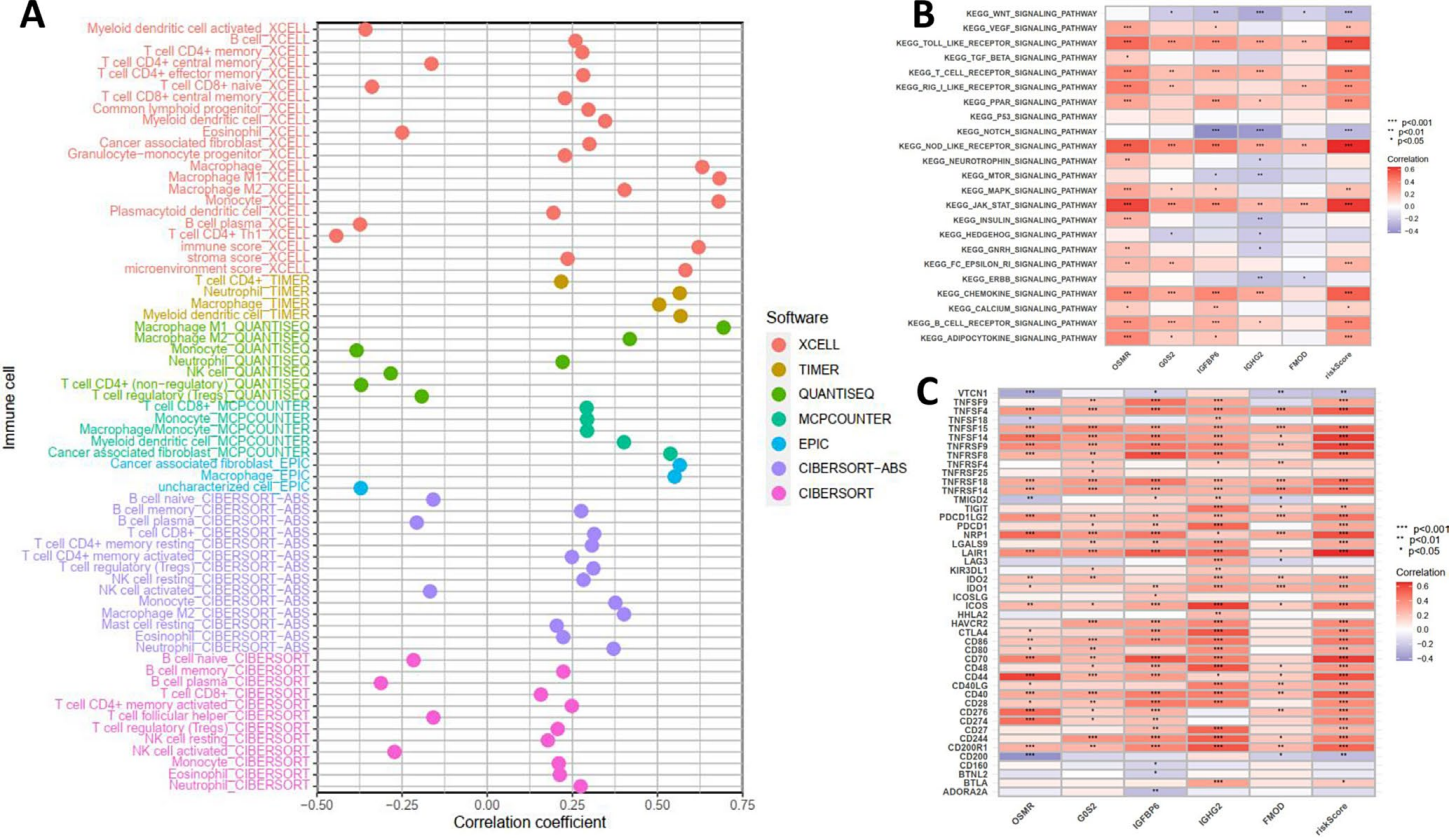
Association of OSMR, G0S2, IGFBP6, IGHG2, FMOD, and their risk model with immune infiltration. (A) Analysis of immune cell enrichment in high‐risk and low‐risk sample groups using software such as XCELL; (B) Enrichment pathway analysis of OSMR, G0S2, IGFBP6, IGHG2, and FMOD and their prognostic risk model; (C) Correlation analysis of OSMR, G0S2, IGFBP6, IGHG2, FMOD, and RS with common immune checkpoints.

Subsequent analysis using various software such as XCELL and TIMER revealed that multiple immune cell infiltrations enriched the prognostic risk model (Figure 3A). GSVA results indicated that OSMR, G0S2, IGFBP6, IGHG2, and FMOD, and the risk model were primarily enriched in pathways such as NOD‐like receptor, Toll‐like receptor, JAK/STAT, and chemokine pathways (Figure 3B), consistent with the previous ferroptosis analysis results (Figure 1F,G). Correlation analysis with common immune checkpoint molecules showed a significant positive correlation between OSMR, G0S2, IGFBP6, IGHG2, FMOD, RS, and most immune checkpoint molecules (Figure 3C). Additionally, by estimating StromalScore, ImmuneScore, and ESTIMATEScore with the ESTIMATE algorithm and assessing their correlation with OSMR, G0S2, IGFBP6, IGHG2, and FMOD, it was evident that the expression of these five genes was significantly correlated with the scores (Figure S5A). Based on ssGSEA, the correlation of OSMR, G0S2, IGFBP6, IGHG2, and FMOD with the proportions of 22 immune cell infiltrations revealed a strong association, with OSMR and G0S2 being most correlated with macrophages, IGHG2 with T cells, and FMOD with Th17 cells (Figure S5B). These results highlight the close relationship between OSMR, G0S2, IGFBP6, IGHG2, FMOD, their risk model, and various immune cell infiltrations.

### 3.3 Analysis of the Expression Distribution of Model Genes OSMR, G0S2, IGFBP6, IGHG2, and FMOD Through Multiomics Approaches

We further explored the expression patterns of the five model genes, OSMR, G0S2, IGFBP6, IGHG2, and FMOD in GBM. Initially, we downloaded several GBM‐related transcriptome RNA sequencing datasets from the GEO database and integrated them with TCGA‐GTEx data. Using a meta‐analysis approach, we validated the differential expression of OSMR, G0S2, IGFBP6, IGHG2, and FMOD. Employing a random‐effects model, the analysis revealed that in tumor tissue samples from GBM patients, OSMR (MD = 1.15, 95% CI = 0.69–1.60), G0S2 (MD = 0.76, 95% CI = 0.24–1.28), IGHG2 (MD = 1.42, 95% CI = 1.14–1.70), and FMOD (MD = 1.48, 95% CI = 0.22–2.74) exhibited significantly higher expression compared to normal control tissue samples. In contrast, IGFBP6 (MD = 0.07, 95% CI = −0.07 to 0.21) showed no statistically significant differential expression between the two groups (Figure S6A–E). Furthermore, sensitivity analysis conducted through a stepwise elimination method indicated consistent results across all groups, confirming the robustness and reliability of the meta‐analysis results (Figure S7A–F). In summary, the mRNA expression of OSMR, G0S2, IGHG2, and FMOD was significantly upregulated in tumor tissue of GBM patients compared to normal control tissue.

Subsequently, we delved into the protein expression differences of OSMR, G0S2, IGFBP6, IGHG2, and FMOD based on the CPTAC and HPA databases. From the CPTAC database, only OSMR, IGFBP6, and FMOD were retrieved, with OSMR protein expression significantly increased in GBM (Figure S8A–C). In the HPA database, only OSMR, IGFBP6, and IGHG2 were found, showing high levels of protein expression in tumor tissue of GBM patients for all three (Figure S8D–F). This suggests that the protein expression of OSMR, IGFBP6, and IGHG2 was significantly elevated in the tumor tissue of GBM patients compared to normal control tissue.

We further utilized GBM‐related scRNA‐seq data GSE206638 from the GEO database to establish a systematic single‐cell transcriptomic map of GBM, aiming to determine the distribution of these genes in the tumor tissue of GBM patients. Initially, we performed quality control and standardized processing of the data using the R package Seurat, filtering out low‐quality cells with nFeature_RNA < 200 & nFeature_RNA > 5000 & percent.mt > 10% (Figure S9A,B). Principal component analysis was conducted on the top 2000 highly variable genes using the RunPCA function, and the top 50 principal components were visualized through the JackStrawPlot function, indicating that all principal components had *p*‐values below 0.05 (Figure S9C). Subsequent analysis with the ElbowPlot function revealed a gradual change in standard deviation around the first 20 principal components without a pronounced inflection point (Figure S9D). A heatmap of the top two principal components was generated using the DimHeatmap function (Figure S9E), displaying the major constituent genes of these components (Figure S9F). Therefore, we proceeded with UMAP clustering analysis using these 20 principal components.

**FIGURE 4:**
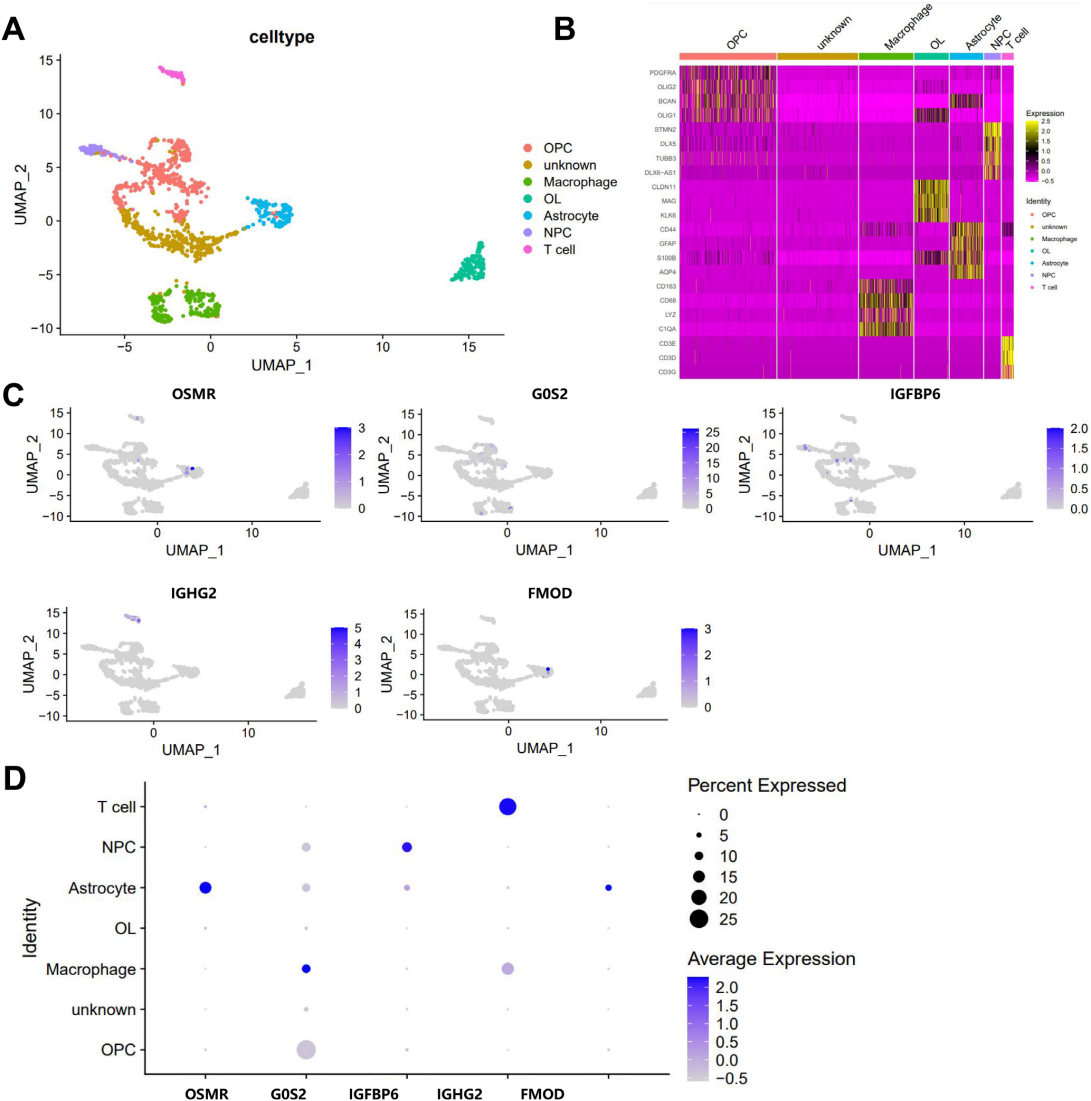
Distribution of five genes in single‐cell transcriptome map of GBM patient tumor tissue samples. (A) Annotation of seven cell clusters into six cell types based on the expression of known marker genes; (B) Heatmap of gene expression in cell markers, where red denotes low expression and yellow denotes high expression; (C) Scatter plot of OSMR, G0S2, IGFBP6, IGHG2, FMOD expression in various cells; (D) Bubble plot of expression of OSMR, G0S2, IGFBP6, IGHG2, FMOD in various cells, with darker colors indicating higher expression levels.

Through UMAP clustering analysis, all cells were classified into seven cell clusters (Figure S9G), eventually identified as oligodendrocyte progenitor‐like cells (OPC), neural progenitor‐like cells (NPC), oligodendrocytes (OL), astrocytes, macrophages, and T cells (Figure 4A). The dot plots and heatmaps of marker genes for each cell type are shown in Figure S9H; in Figure 4B, PDGFRA, OLIG2, BCAN, and OLIG1 were identified as marker genes for OPC, while STMN2, DLX5, TUBB3, and DLX6‐AS1 were found to be marker genes for NPC. Additionally, CLDN11, MAG, and KLK6 were identified as marker genes for oligodendrocytes, whereas CD44, GFAP, S100B, and AQP4 were markers for astrocytes. Marker genes for macrophages included CD163, CD68, LYZ, and C1QA; those for T cells were CD3E, CD3D, and CD3G. However, Cluster1 lacked discernible marker genes, making its classification unknown.

To investigate the role of the five genes (OSMR, G0S2, IGFBP6, IGHG2, and FMOD) in specific cells within the tumor tissue of GBM patients, we illustrated the distribution of these genes across different cell types. The results indicated that in GBM tumor tissue, OSMR and FMOD were primarily distributed in astrocytes, IGFBP6 in NPC, G0S2 in macrophages, and IGHG2 in T cells (Figure 4C,D). These findings align with previous analyses on the TME and immune infiltration, emphasizing the close association of ferroptosis subtyping and the risk model with immune cells such as macrophages. Combining the results of scRNA‐seq analysis, we further confirmed the correlation of OSMR, G0S2, IGFBP6, IGHG2, FMOD, and their risk model with macrophages and other immune infiltrations, providing new theoretical evidence for elucidating the specific mechanisms by which ferroptosis impacts tumor immunity in GBM.

### 3.4 Knockdown of OSMR Suppresses the Growth of GBM and Inhibits Ferroptosis in GBM

Both astrocytes and NPC (neural progenitor‐like cells) possess regenerative potential and are potential sources of glioma cells [68]. In our bioinformatics analysis, we observed that OSMR and FMOD were mainly distributed in astrocytes in GBM tumor tissue, while IGFBP6 was predominantly found in NPC cells, all of which are crucial factors in GBM pathogenesis. Thus, we initially assessed the expression levels of these genes in normal human astrocytes (HA), human GBM cell lines LN229, BT325, A172, and murine GBM cells GL261. The results revealed significant upregulation of OSMR, FMOD, and IGFBP6 in LN229, BT325, A172, and GL261 cells compared to HA cells, with OSMR showing the most notable increase in expression (Figure S10A). OSMR has been reported to regulate oxidative stress levels in glioma stem cells and increase GBM resistance to ionizing radiation [48]. Furthermore, OSMR is associated with the malignant transformation of GBM [69]. Survival analysis based on the TCGA‐GBM dataset demonstrated that patients with high OSMR expression had poorer OS, disease‐specific survival, and progression‐free interval than those with low OSMR expression (Figure S10B–D). Hence, we selected OSMR as the target for further study.

Subsequent Western blot experiments revealed a significant increase in OSMR protein levels in LN229, BT325, A172, and GL261 cells compared to normal astrocytes (HA) (Figure S10E). We chose LN229 and GL261 cell lines for creating OSMR knockdown (KD) cell lines through lentiviral infection. RT‐qPCR and Western blot demonstrated that OSMR was markedly downregulated in OSMR‐KD#1 or OSMR‐KD#2 cells compared to the OSMR‐NC group, confirming the successful establishment of the OSMR‐KD cell line for further experiments (Figure S10F,G). These results indicate a significant increase in OSMR expression in GBM.

**FIGURE 5:**
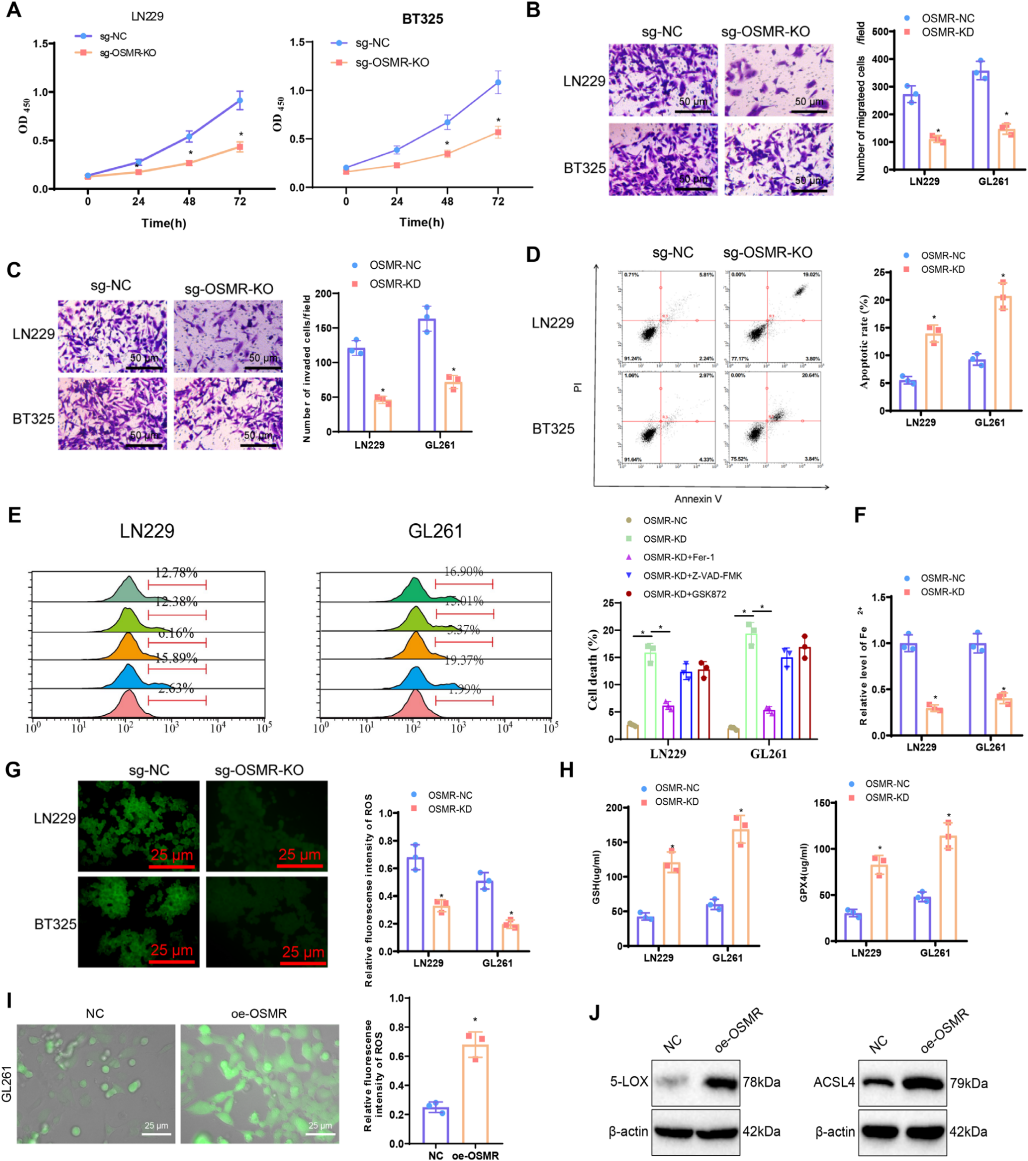
Validation of OSMR function in vitro cell experiments. (A) CCK8 assay to measure proliferation ability of cells in each group; (B, C) Transwell assay to assess migration and invasion capacity of cells in each group (scale bar = 50 μm); (D) Flow cytometry analysis of the apoptotic ability of cells in each group; (E) Treatment of OSMR‐KD infected cells with 1 μM Fer‐1, 20 μM pan‐caspase inhibitor Z‐VAD‐FMK, or 10 μM RIPK3 inhibitor GSK872 for 30 h, followed by SYTOX Green staining and flow cytometry to detect cell death; (F) Iron ion assay kit to measure Fe^2+^ enrichment in cells in each group; (G) Immunofluorescence detection of ferroptosis‐related indicator ROS levels in cells of each group; (H) ELISA to measure ferroptosis‐related indicators GSH and GPX4 levels in cells of each group; (I) Immunofluorescence detection of iron death‐related indicators ROS levels in GL261 cells overexpressing OSMR; (J) Expression of LOX and ACSL4 detected in GL261 cells overexpressing OSMR protein using Western Blot. *p < 0.05, all cell experiments were independently repeated three times.

To further validate the impact of OSMR on GBM growth, cell proliferation assays using CCK8 showed a significant decrease in proliferation capacity in OSMR‐KD cells compared to OSMR‐NC cells (Figure 5A). Similarly, transwell assays indicated a decreased invasion and migration capacity in OSMR‐KD cells compared to controls (Figure 5B,C).

Flow cytometry analysis of cell death in OSMR‐KD cells showed a notable increase in cell death compared to the OSMR‐NC group (Figure 5D). Moreover, to determine the type of induced cell death, specific inhibitors (including Fer‐1 for ferroptosis, Z‐VAD‐FMK for necrosis, and GSK872 for apoptosis) were preincubated after OSMR knockdown. Flow cytometry analysis of cell death revealed that Fer‐1 significantly reversed the cell death induced by OSMR‐KD, while the effects of Z‐VAD‐FMK and GSK872 were limited (Figure 5E). We further investigated the impact of OSMR on GBM cell ferroptosis. Ferroptosis is characterized by increased iron accumulation, lipid peroxidation, elevated ROS levels, and altered gene expression in iron homeostasis and lipid peroxidation metabolism [70]. The results demonstrated a significant decrease in iron accumulation in OSMR‐KD cells compared to OSMR‐NC cells (Figure 5F). ROS levels were significantly lower in OSMR‐KD cells (Figure 5G), and GSH and GPX4 levels were notably higher in OSMR‐KD cells than in OSMR‐NC cells, as evidenced by ELISA measurements (Figure 5H). We further elucidated the regulatory role of OSMR in the ferroptosis pathway in glioma. By integrating the latest literature and experimental results [71], we provided a detailed explanation of the key role of OSMR in iron metabolism and lipid peroxidation, which are central components of the ferroptosis process. Specifically, our study showed that OSMR directly affects the accumulation and distribution of iron ions by regulating the expression of genes related to intracellular iron metabolism. The occurrence of ferroptosis depends on elevated levels of free iron within the cell, which leads to the generation of ROS and, subsequently, the initiation of lipid peroxidation. Our data demonstrated that OSMR overexpression significantly increased intracellular iron ion concentration, promoted ROS production, and ultimately exacerbated lipid peroxidation (Figure 5I). Therefore, OSMR plays a critical role in regulating iron metabolism pathways, creating conditions for ferroptosis induction. Additionally, OSMR is involved in lipid metabolic pathways, particularly in regulating the oxidation of polyunsaturated fatty acids (PUFAs). Our results showed that OSMR overexpression enhanced the expression of key lipid peroxidation enzymes such as ACSL4 and LOX, which are crucial steps in the ferroptosis process (Figure 5J).

In in vitro experiments, we established a stable GL261 glioma cell line overexpressing OSMR. First, by transfecting OSMR overexpression plasmids, we successfully elevated OSMR expression levels in glioma cells (Figure S11A). Subsequently, using the EdU cell proliferation assay kit and colony formation assay (Figure S11B,C), we assessed the impact of OSMR overexpression on glioma cell proliferation. The results showed that, compared to the control group, OSMR overexpression significantly promoted cell proliferation rates, and in the colony formation assay, the number of colonies formed by OSMR‐overexpressing cells was markedly increased. These findings demonstrate that OSMR plays a promoting role in the proliferation of glioma cells.

These findings indicate that knocking down OSMR can inhibit GBM growth and suppress GBM ferroptosis.

### 3.5 Knockdown of OSMR Alleviates Suppression of CD8 ^+^ T Cell Activity While Influencing Tumor‐Associated Macrophage Polarization

**FIGURE 6:**
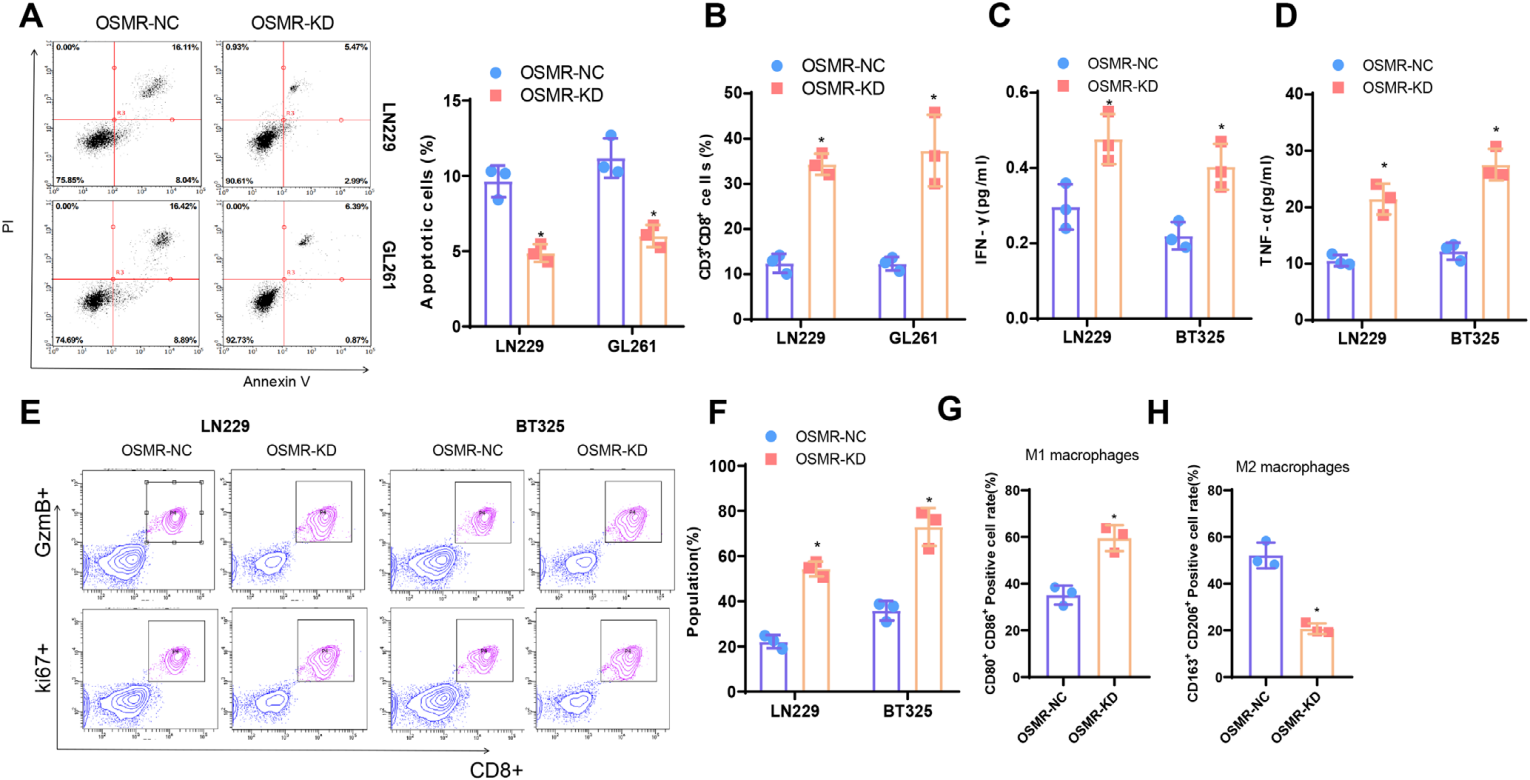
Impact of OSMR knockdown on CD8^+^ T cell activity. (A) Flow cytometry analysis of apoptosis in LN229 and GL261 cells co‐cultured with CD8^+^ T cells; (B) Flow cytometry assessment of the activity of CD8^+^ T cells co‐cultured with LN229 and GL261 cells; (C, D) ELISA analysis of TNF‐α and IFN‐γ concentrations in the co‐culture system; (E, F) Flow cytometry examination of Ki67 and GzmB positive cell numbers after co‐culturing LN229, GL261 cells with CD8^+^ T cells; (G, H) Flow cytometry to assess macrophage polarization markers (CD80/CD86 for M1 macrophages and CD163/CD206 for M2 macrophages) in THP‐1 cells co‐cultured with OSMR‐NC and OSMR‐KD cells. *p < 0.05 compared to the OSMR‐NC group; all cell experiments were independently repeated three times.

We further validated the impact of OSMR downregulation on GBM, particularly on CD8^+^T cell activity. Co‐culturing OSMR‐KD and OSMR‐NC GBM cells with CD8^+^T cells resulted in increased induction of GBM cell apoptosis by CD8^+^ cytotoxic T cells in the presence of OSMR‐KD (Figure 6A). Flow cytometry analysis revealed enhanced activity of CD8^+^T cells in the OSMR‐KD group (Figure 6B). In the co‐culture system of OSMR‐KD Huh7 and SNU‐449 cells with CD8^+^T cells, there was an increase in the production of TNF‐α and IFN‐γ (Figure 6C,D). Moreover, the OSMR‐KD group exhibited significantly enhanced proliferation and cytotoxicity of CD8^+^T cells, as evidenced by increased numbers of Ki67 and GzmB‐positive cells (Figure 6E,F). These findings suggest that downregulating OSMR can mitigate the inhibitory effect on CD8^+^ T cell activity. Since macrophages are the most infiltrated cells in glioblastoma tissues, we co‐cultured OSMR‐KD and OSMR‐NC glioblastoma cells with THP‐1 cells (THP‐1 differentiated macrophages [72]). Subsequently, macrophage polarization markers CD80/CD86 (M1 macrophages) and CD163/CD206 (M2 macrophages) were detected via flow cytometry [73]. The results (Figure 6G,H) showed that the absence of OSMR in tumor cells promoted an increase in the proportion of M1 macrophages and decreased the positive proportion of anti‐inflammatory M2 macrophages.

### 3.6 Downregulation of OSMR Inhibits GBM Growth in Vivo

**FIGURE 7:**
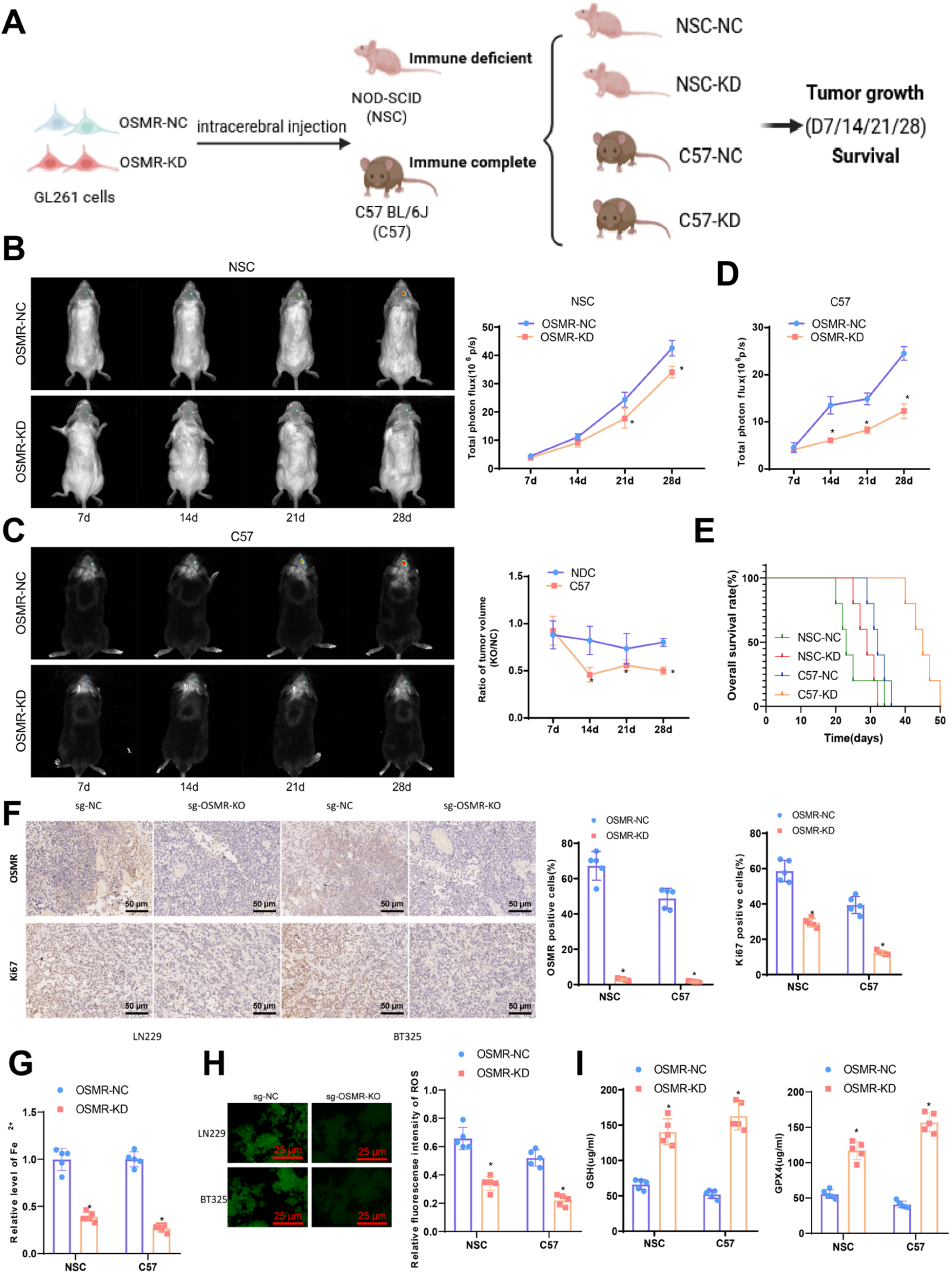
Further confirmation of the key role of OSMR in in vivo animal experiments. (A) Investigation of OSMR's function in immunodeficient (NOD‐SCID, NSC) and immunocompetent (C57BL/6J, C57) tumor‐bearing mice; (B, C) Representative pseudocolored bioluminescence images and quantitative analysis of bioluminescence signal intensity on days 7, 14, 21, and 28 in NSC (B) and C57 (C) mice implanted with OSMR‐NC or OSMR‐KD GL261 cells; (D) Photon flux signal intensity ratio of OSMR‐KD and OSMR‐NC treated NSC and C57 mice groups; (E) Kaplan–Meier OS curves in NSC and C57 mice postimplantation of OSMR‐NC or OSMR‐KD GL261 cells; (F) IHC staining to determine the number of OSRM and Ki67 positive cells in transplanted tumors (scale bar = 50 μm); (G) Iron ion assay kit to measure Fe^2+^ accumulation in transplanted tumors; (H) Immunofluorescence analysis of ferroptosis‐related indicator ROS levels in transplanted tumors; (I) Enzyme‐linked immunosorbent assay (ELISA) to evaluate ferroptosis‐related indicators GSH and GPX4 levels in cerebrospinal fluid (CSF). *p < 0.05 compared to the OSMR‐NC group, five mice per group.

In order to elucidate the role of OSMR in regulating immune responses, we utilized two in situ mouse models using GL261 mouse glioma cells. OSMR downregulated GL261 cells infected with lentivirus were implanted into immunodeficient NOD‐SCID (NSC) or immunocompetent C57BL/J6 (C57) mice (Figure 7A). Tumor growth was assessed using bioluminescent imaging, and the survival time of tumor‐bearing mice was recorded. Compared to the OSMR‐NC group, significant inhibition of tumor growth was observed in NSC mice with OSMR‐KD at day 21 and day 28 (Figure 7B). Similarly, the tumor growth rate was slower in C57 mice treated with OSMR‐KO compared to the OSMR‐NC group (Figure 7C). Importantly, OSMR downregulation exhibited a more pronounced suppression of tumor growth in immunocompetent C57 mice compared to immunodeficient NSC mice, with a lower tumor volume ratio in the KO/NC group (Figure 7D). Furthermore, tumor‐bearing mice in the OSMR‐KD group showed significantly prolonged survival in both NSC (37 and 32 days, *p* = 0.0273) and C57 mice (56 and 40 days, *p* = 0.0039) compared to the OSMR‐NC group (Figure 7E). Notably, OSMR deficiency had a more pronounced effect in extending immunocompetent mice's survival than immunodeficient mice (Figure 7F). IHC staining revealed that OSMR‐KD tumors had almost undetectable OSMR‐positive cells and a significant decrease in Ki67‐positive cells compared to the OSMR‐NC group, indicating reduced proliferative activity of OSMR‐KD tumors. Additionally, we investigated the impact of OSMR on tumor ferroptosis in vivo. Iron ion assay kit results demonstrated significantly reduced iron ion accumulation in OSMR‐KD cells compared to the OSMR‐NC group (Figure 7G). Subsequent ROS detection revealed a significant decrease in ROS levels in tumor tissues from the OSMR‐KD group compared to the OSMR‐NC group (Figure 7H). ELISA analysis showed a significant increase in GSH and GPX4 levels in the cerebrospinal fluid of the OSMR‐KD group compared to the OSMR‐NC group (Figure 7I). In vivo experiments, we also injected OSMR‐overexpressing GL261 cells into a mouse model to simulate glioblastoma tumor formation. By monitoring changes in tumor volume, we observed that tumors overexpressing OSMR grew significantly faster compared to the control group (Figure S12A). Additionally, histopathological analysis confirmed that the expression level of the proliferation marker Ki‐67 in tumor tissues was markedly increased (Figure S12B), which was consistent with the in vitro results, indicating that OSMR overexpression enhanced the proliferative capacity of glioma cells. These results indicate that the downregulation of OSMR can inhibit GBM growth in vivo by promoting ferroptosis.

### 3.7 OSMR Deficiency Suppresses Tumor Growth by Rescuing Anti‐Tumor Immune Response

**FIGURE 8:**
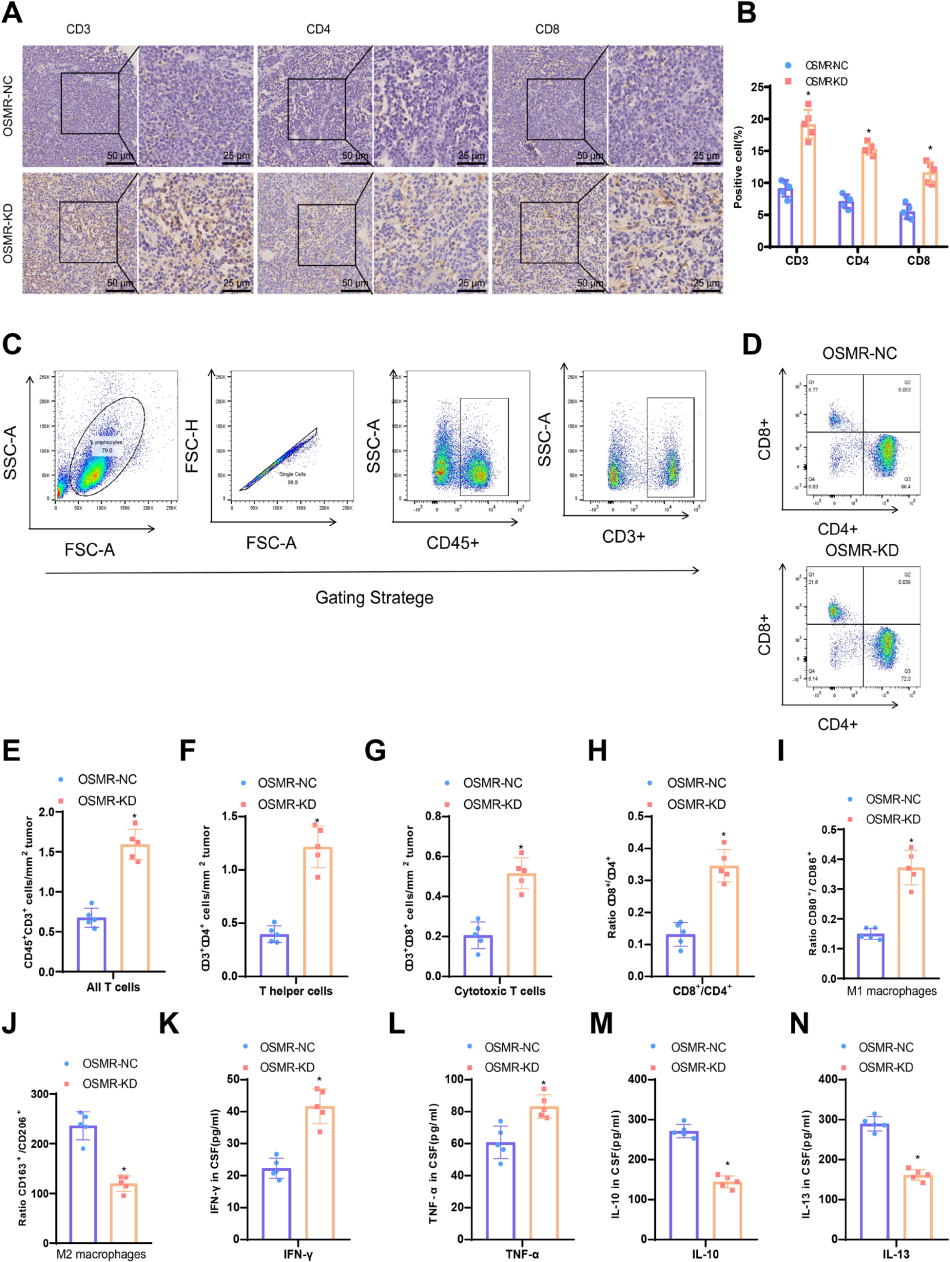
Enhanced recruitment and infiltration of T cells in vivo due to OSMR deletion. (A) Representative IHC staining images of CD3^+^, CD4^+^, and CD8^+^ T cells in tumors 28 days postimplantation of OSMR‐NC and OSMR‐KD GL261 cells; (B) Quantitative analysis of the number of positive cells in each field of the IHC staining images, as shown in panel A; (C) Flow cytometry gating strategy to analyze tumor infiltration of CD45^+^CD3^+^CD4^+^CD8^−^ (CD4^+^) T helper cells and CD45^+^CD3^+^CD4^−^CD8^+^ (CD8^+^) cytotoxic T cells in mouse GBM tissues; (D) Representative flow cytometry images of tumor‐infiltrating CD4^+^ and CD8^+^T cells. FACS quantification of immune cells, including (E–H) CD3^+^ T cells, (F) CD4^+^ T cells, (G) CD8^+^ T cells, and (H) the ratio of CD8^+^/CD4^+^ T cells; (I, J) Flow cytometry to assess macrophage polarization markers (CD80/CD86 for M1 macrophages and CD163/CD206 for M2 macrophages) in OSMR‐NC and OSMR‐KD group mice; (K–N) ELISA assessment of concentrations of (K) IFN‐γ, (L) TNF‐α, (M) IL‐10, and (N) IL‐13 in the cerebrospinal fluid of OSMR‐NC and OSMR‐KD mice on day 28. *p < 0.05 compared to the OSMR‐NC group, five mice per group.

Given the more significant reduction in tumor growth and improved survival rates in C57 mice with OSMR‐deficient GBM compared to NSC mice, we hypothesized that OSMR may influence the anti‐tumor function of the immune system. As T cells play a crucial role in anti‐tumor immune responses, we conducted immunohistochemical analysis in mouse GBM tissues. IHC staining showed a significant increase in infiltrating CD3^+^, CD4^+^, and CD8^+^T cells in OSMR‐deficient tumors (Figure 8A,B). Consistent with the IHC results, flow cytometry of GBM tissues showed that knocking down OSMR significantly increased the number of infiltrating T cells (Figure 8C,D), including total T cells (CD45^+^CD3^+^) (Figure 8E), T‐helper cells (CD45^+^CD3^+^CD4^+^CD8^−^) (Figure 8F), and cytotoxic T cells (CD45^+^CD3^+^CD4^−^CD8^+^) (Figure 8G). Particularly, the deficiency of OSMR increased the ratio of cytotoxic T cells (CD4^−^CD8^+^) to T helper cells (CD4^−^CD8^+^) (Figure 8H). The flow cytometry results of macrophages also showed that the absence of OSMR increased the proportion of M1 macrophages and decreased the proportion of M2 macrophages (Figure 8I,J). Furthermore, at 28 days postinjection, enzyme‐linked immunosorbent assay (ELISA) was used to assess pro‐inflammatory and immunosuppressive cytokines in the cerebrospinal fluid (CSF). The results showed that OSMR deficiency significantly increased the levels of the anti‐tumor immune cytokines IFN‐γ and TNF‐α (Figure 8K,L). In contrast, IL‐10 and IL‐13, which are associated with the suppression of anti‐tumor immune function, showed statistically significant decreases (Figure 8M,N).

## 4 Discussion

In previous studies, ferroptosis has been regarded as a form of cell death associated with iron metabolism disruption in organisms, holding significant research value in tumor biology [9, 15, 16, 74]. However, in the research of GBM, the issue of utilizing ferroptosis for disease classification and prognosis assessment remains inadequately investigated [75]. This study conducted an in‐depth investigation on FRGs from databases such as FerrDB and utilized the TCGA and CGGA databases as training and testing cohorts, successfully categorizing GBM patients based on FRGs. The results indicate that this classification method holds significant value in prognosis evaluation, marking a key innovation of this study and distinguishing it from previous research [76].

Furthermore, this study further explored the correlation between ferroptosis classification and GBMTME and immune infiltration. It was found that there were significant differences in stromal and immune scores, as well as immune cell content, between the two categories (cluster A and cluster B). This outcome suggests a close association of ferroptosis classification with GBMTME and immune infiltration, enhancing the depth and breadth of understanding and application of ferroptosis classification. Compared to past studies, this research offers a fresh perspective on the relationship between TME and immune infiltration [77]. Additionally, prior studies have demonstrated the involvement of glial cells in neurogenesis or glioma genesis in the peritumoral or tumor marginal tissues. By contrasting glial cells with glioma cells, this study provides theoretical support on how OSMR influences GBM progression in different cellular environments. The dual regulatory mechanism of OSMR in the ferroptosis pathway: By influencing iron metabolism and lipid peroxidation, OSMR promotes the occurrence of ferroptosis in glioblastoma. This finding offers a new perspective for understanding the pathological mechanisms of glioblastoma and suggests the potential application of OSMR as a therapeutic target related to ferroptosis.

Among 342 screened differentially expressed genes, this study successfully constructed a risk model composed of five genes (OSMR, G0S2, IGFBP6, IGHG2, and FMOD) through single‐factor COX and LASSO regression analyses. This model was validated in CGGA data and could accurately assess the prognosis of GBM patients. Compared to previous prognostic models, this study's model encompasses various genes that have not received sufficient attention as key prognostic factors in other studies [78]. This innovative model construction method provides a new research avenue for future prognosis evaluations.

Moreover, through meta‐analysis and protein expression data analysis, this study conducted an in‐depth examination of these five genes, revealing significant upregulation of the mRNA of these genes and partial protein expression in GBM patient tumor tissues compared to normal control tissues. This finding partially unveils the potential crucial roles these genes may play in the occurrence and development of GBM, offering a potential biomarker.

By conducting scRNA‐seq analysis, this study explicitly illustrates the distribution of key genes in various cells, which holds significant importance in further understanding the specific roles and possible mechanisms of these genes in GBM. The innovative aspect of this study lies in integrating single‐cell sequencing data, enabling a more precise understanding of the expression and functions of these genes at the cellular level.

Based on the aforementioned results, the preliminary conclusion drawn is that a risk model accurately predicting the prognosis of GBM patients was constructed based on FRGs. This model consists of OSMR, G0S2, IGFBP6, IGHG2, and FMOD, with the mRNA or protein expression of these five genes significantly upregulated in GBM patient tumor tissues, closely associated with immune cell infiltration such as macrophages. GBM is a centrally nervous system tumor with extremely poor clinical prognoses [1, 2, 3, 4, 79]. Despite improvements in survival periods for GBM patients under comprehensive treatments like surgery, radiotherapy, and chemotherapy, the majority of patients still have survival periods of < 2 years [80]. Therefore, researching new biomarkers and discovering novel therapeutic targets is crucial for enhancing the survival prognosis of GBM patients. As a new form of cell death, ferroptosis has been proven to play a critical role in various types of tumors, including GBM [8, 65, 81].

This study established a risk model comprising OSMR, G0S2, IGFBP6, IGHG2, and FMOD based on ferroptosis classification and verified the value of this model in predicting the survival prognosis of GBM patients. This is of significant reference value for clinical prognosis assessment and the formulation of individualized treatment strategies. Furthermore, through scRNA‐seq, the expression distribution of these key genes in different cells was revealed, aiding in a deeper understanding of the functions and mechanisms of these genes in GBM.

First, although this study utilized public databases for research, the sample sizes in these databases are limited, and there might be sample biases. Additionally, further prospective clinical studies are required to validate this risk model. Second, this study mainly focused on the roles of FRGs in GBM, but ferroptosis is a complex BP oligodendrocyte progenitor‐like cell (OPC) influenced by various factors; therefore, further research is needed to explore other potential factors affecting GBM prognosis. Lastly, while scRNA‐seq can reveal the gene expression status in individual cells, it cannot provide information on spatial connections between cells, one of its limitations. Despite some limitations, this study provides a new perspective for a deeper understanding of the pathophysiological mechanisms and disease progression of GBM. Future research can further verify the expression and distribution of these key genes in GBM patient tissues through techniques such as tissue chips or IHC. Additionally, although this risk model shows certain value in predicting the survival prognosis of GBM patients, further validation through more prospective clinical trials is needed. Furthermore, these key genes and their related signaling pathways may serve as novel therapeutic targets, warranting further exploration of their potential applications in GBM treatment. Additionally, while this study determined the impact of OSMR on GBM growth and metastasis through in vivo and in vitro experiments, the specific molecular regulatory mechanism remains unknown, as well as the role played by immune cells, which is the focus of future research.

## Author Contributions

Yaqiu Wu and Ling Liu contributed equally to the conception and design of the study. Zhili Li performed data collection and statistical analyses. Tian Zhang contributed to the multiomics analysis and interpretation of the results. Qi Wang and Meixiong Cheng supervised the project and provided critical revisions to the manuscript. All authors participated in drafting the manuscript, approved the final version, and are accountable for all aspects of the work.

## Ethics Statement

All animal experiments were approved by the Animal Ethics Committee of Sichuan Provincial People's Hospital.

## Conflicts of Interest

The authors declare no conflicts of interest.

## Supporting information


Figure S1

Figure S2

Figure S3

Figure S4

Figure S5

Figure S6

Figure S7

Figure S8

Figure S9

Figure S10

Figure S11

Figure S12

Table S1
Table S2
Table S3

## Data Availability

All data can be provided as needed.
