# Supplementary figures and images for: A Risk Model Based on Ferroptosis‐Related Genes OSMR, G0S2, IGFBP6, IGHG2, and FMOD Predicts Prognosis in Glioblastoma Multiforme
Source: CNS Neurosci Ther. 2025 Jan 15;31(1):e70161. doi: 10.1111/cns.70161 (PMC11735466; doi:10.1111/cns.70161)

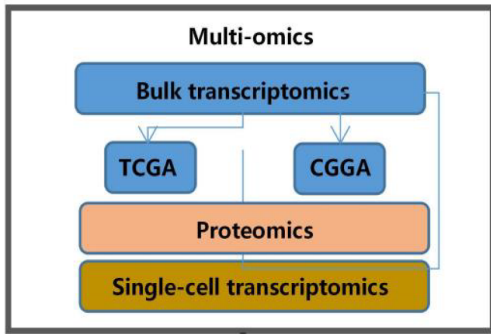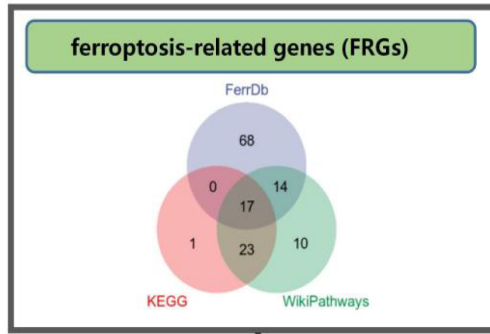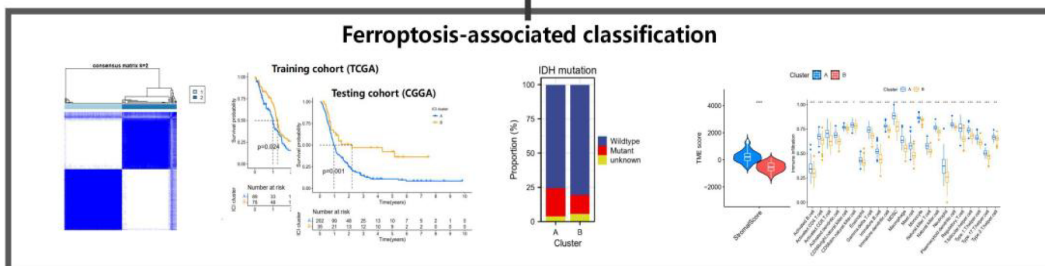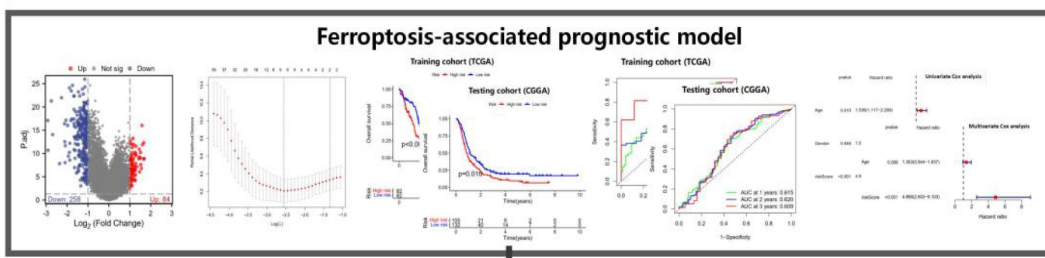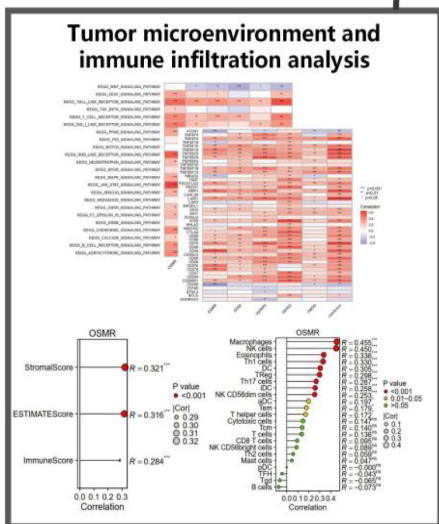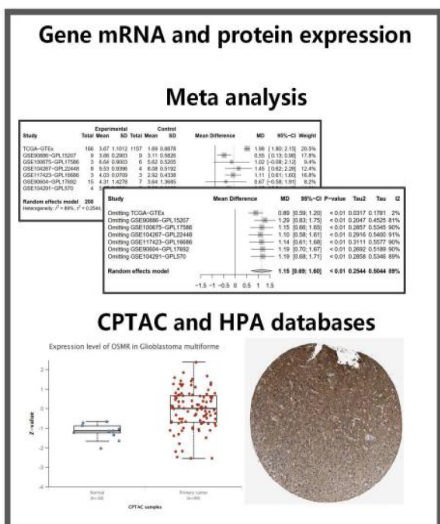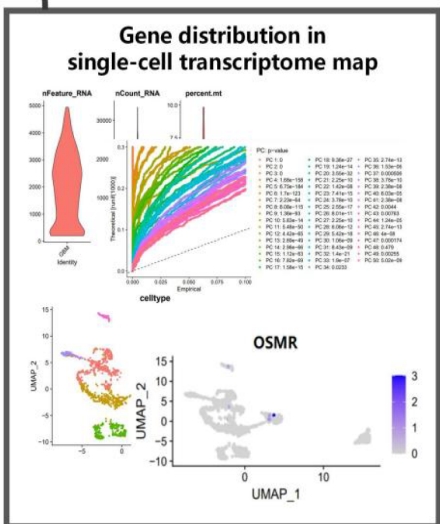

**OSMR**

**Experimental verification in vitro and in vivo**

Supplement: Supplementary file 1 — Figure S1 [file CNS-31-e70161-s010.pdf]

**A**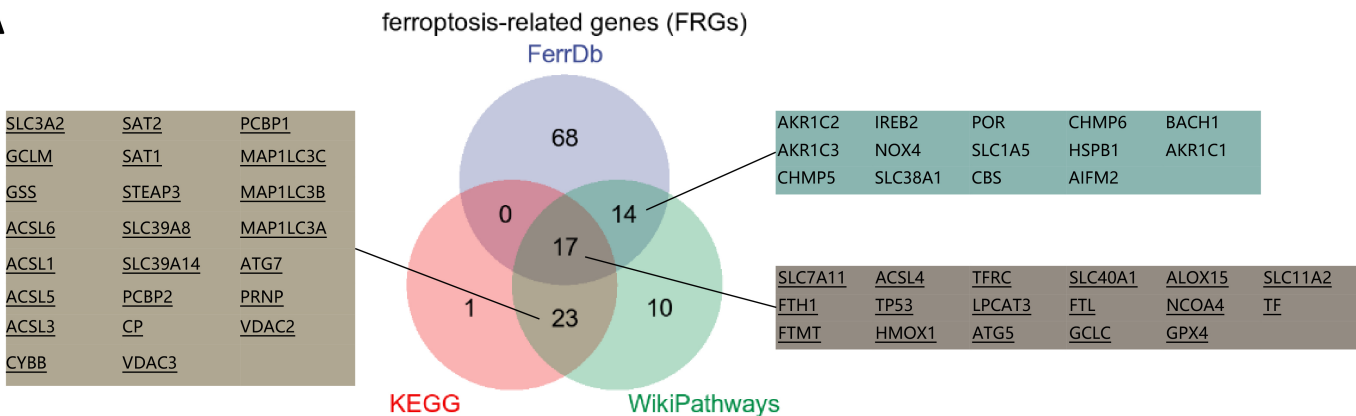**B**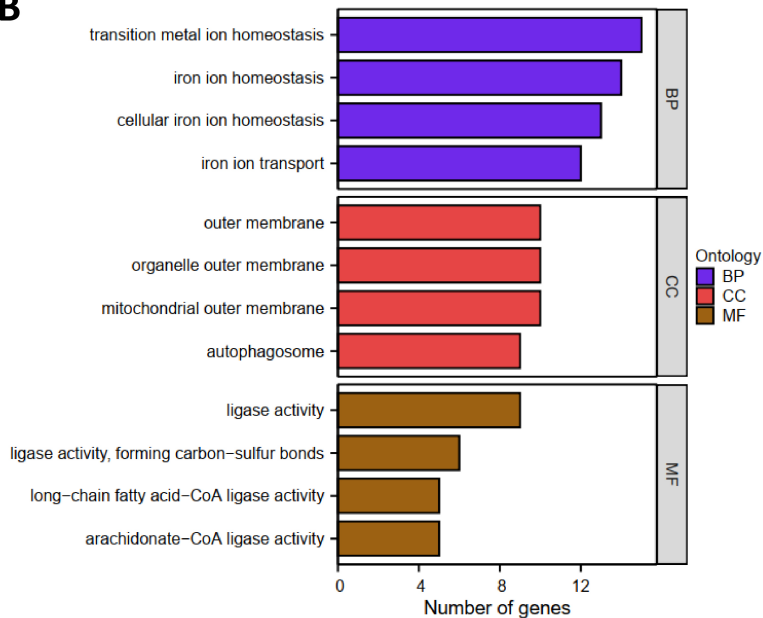**C**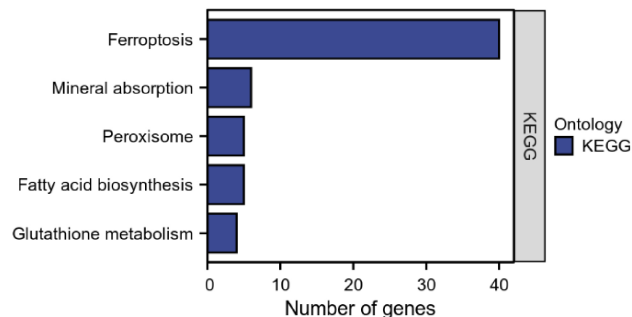

Supplement: Supplementary file 2 — Figure S2 [file CNS-31-e70161-s009.pdf]

**A**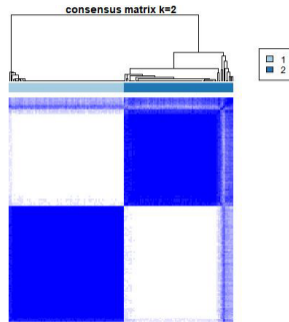**B**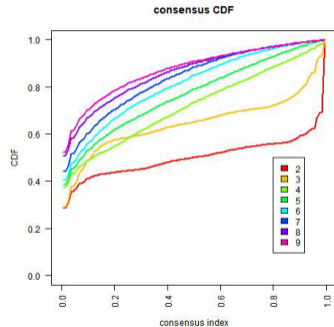**C**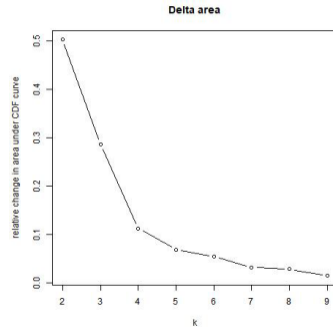**D**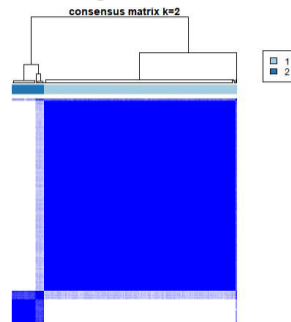

Supplement: Supplementary file 3 — Figure S3 [file CNS-31-e70161-s012.pdf]

**A**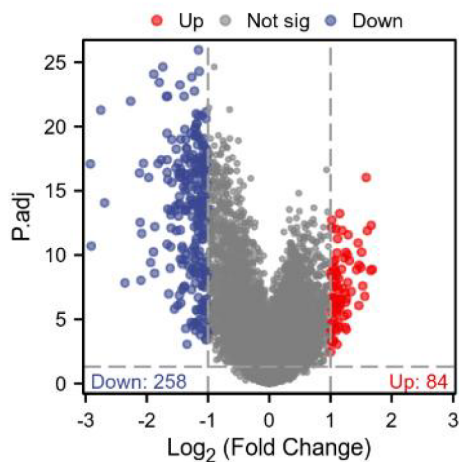**B**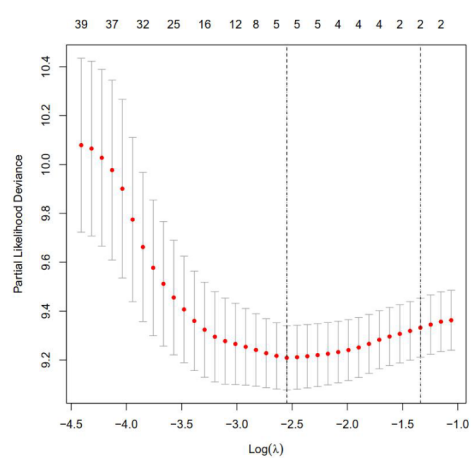**C**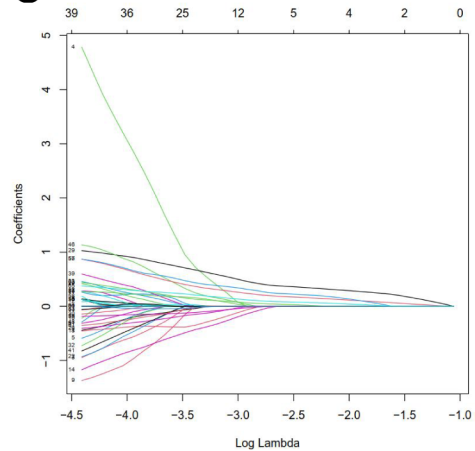**D**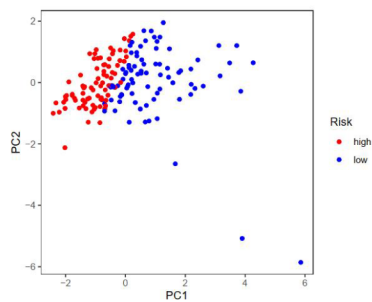**E**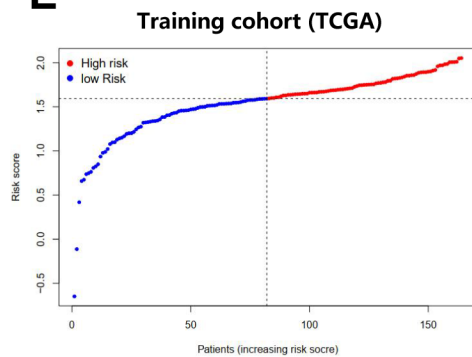**F**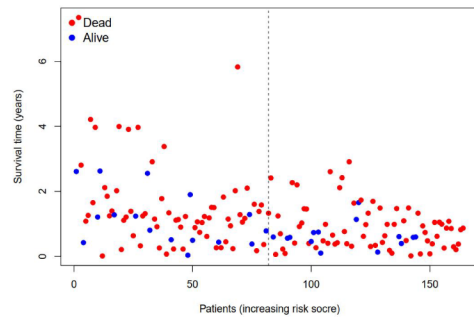**G**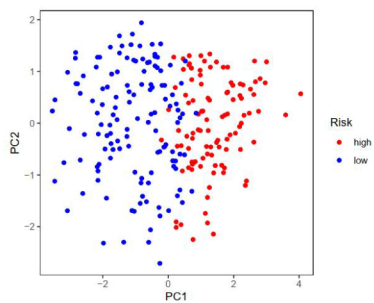**H**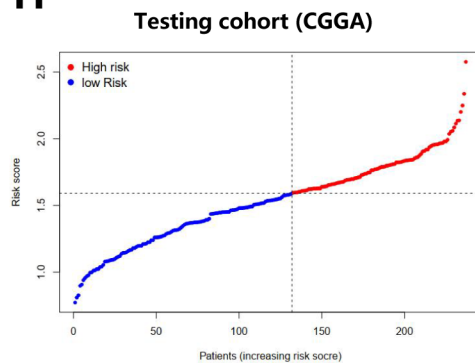**I**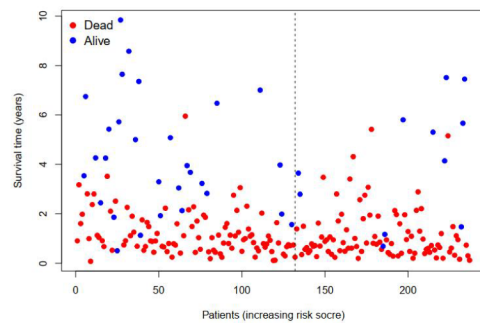

Supplement: Supplementary file 4 — Figure S4 [file CNS-31-e70161-s004.pdf]

**A**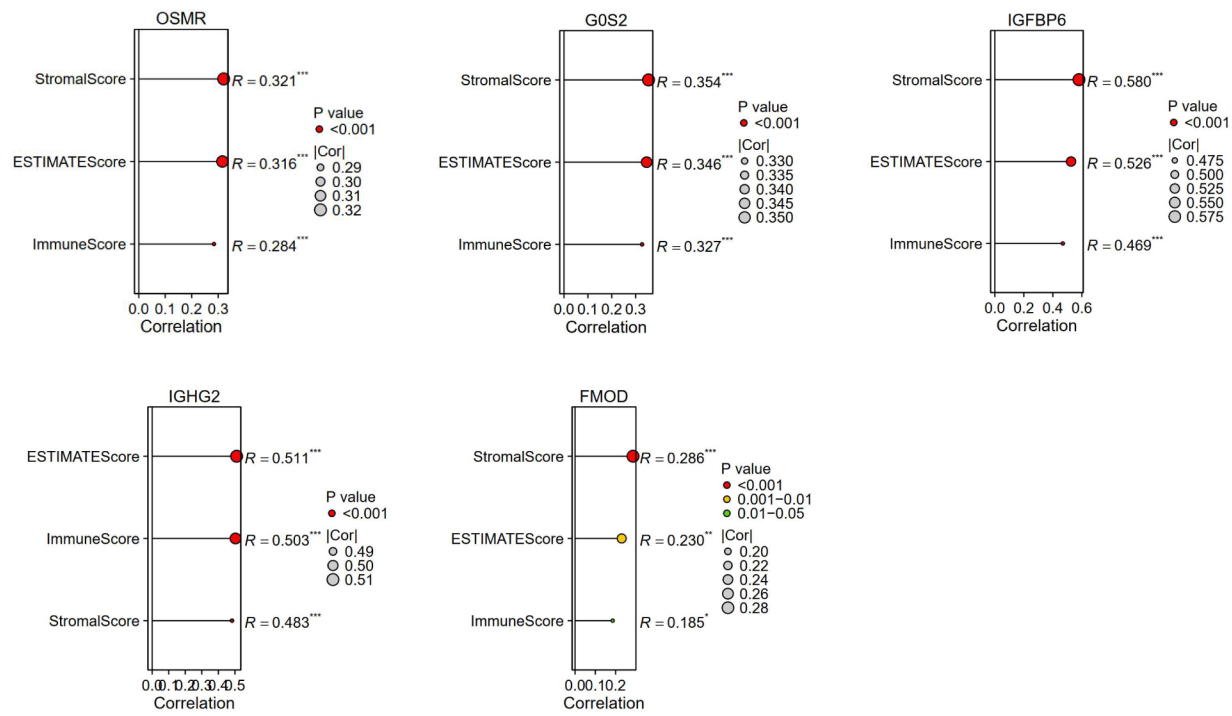**B**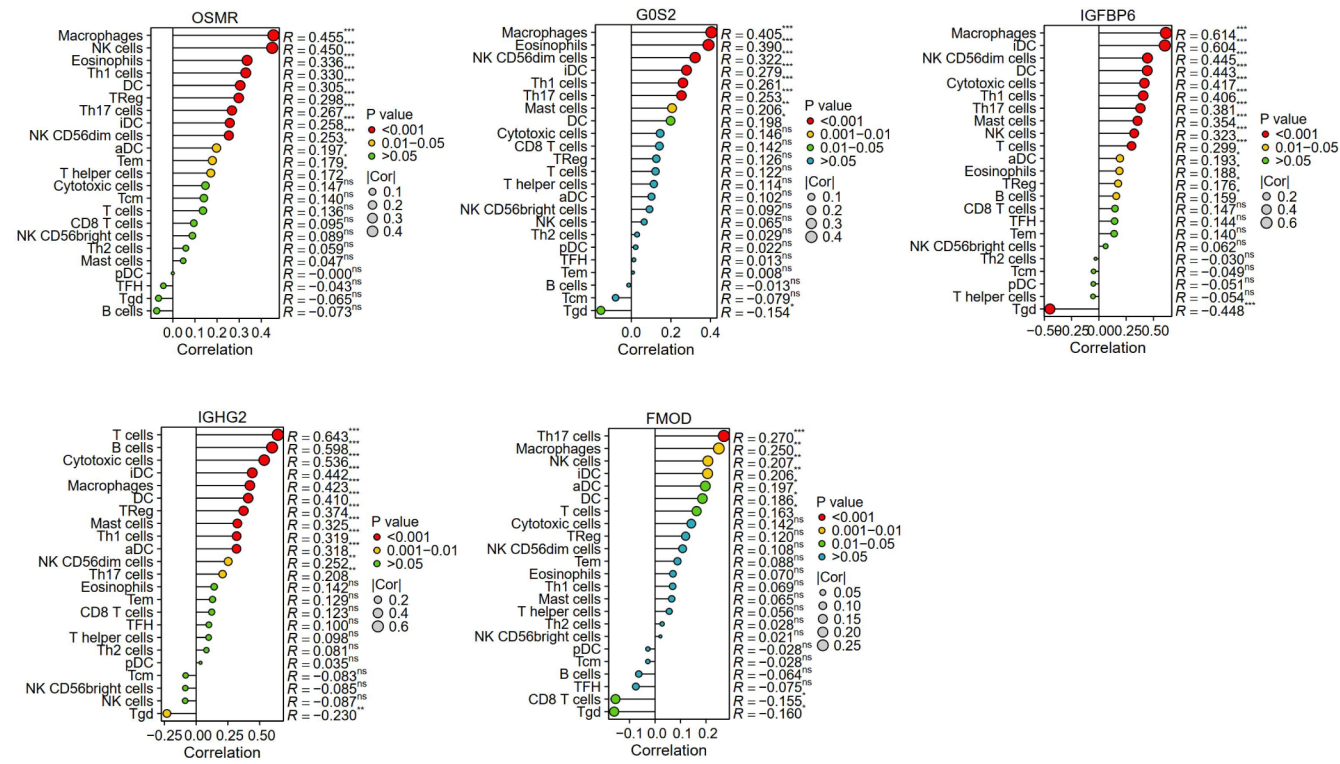

Supplement: Supplementary file 5 — Figure S5 [file CNS-31-e70161-s013.pdf]

**A****OSMR**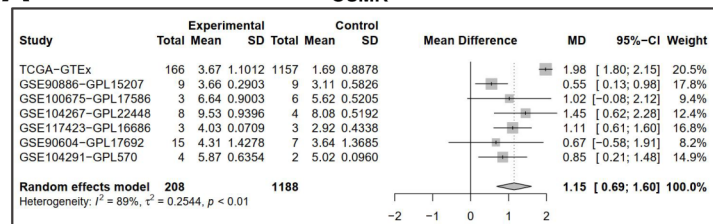**B****G0S2**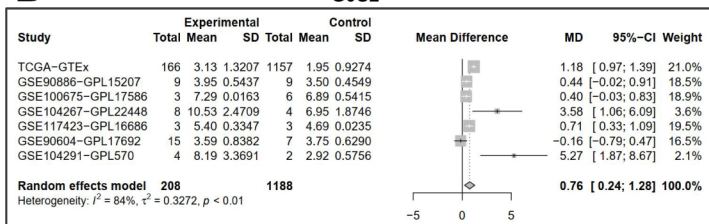**C****IGFBP6**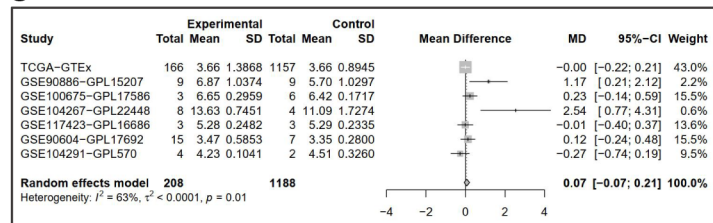**D****FMOD**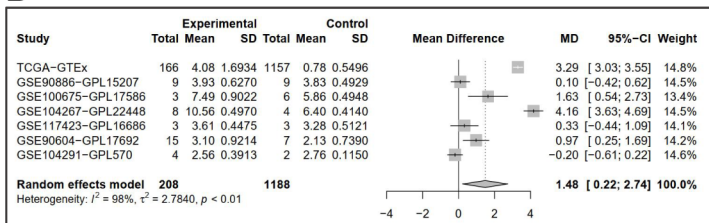**E****IGHG2**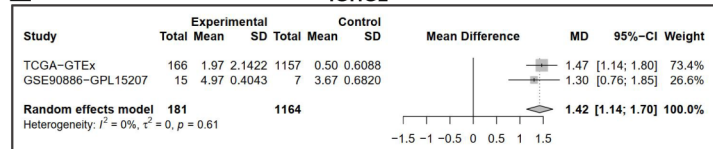

Supplement: Supplementary file 6 — Figure S6 [file CNS-31-e70161-s008.pdf]

**A****OSMR**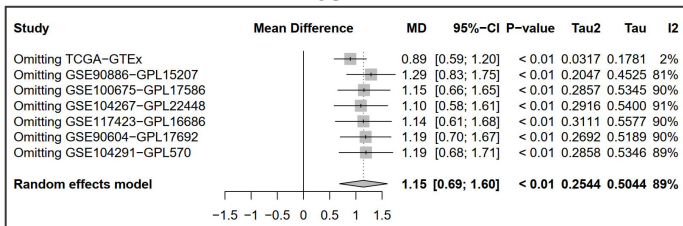**B****G0S2**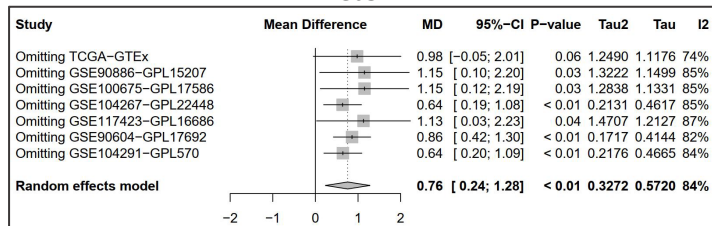**C****IGFBP6**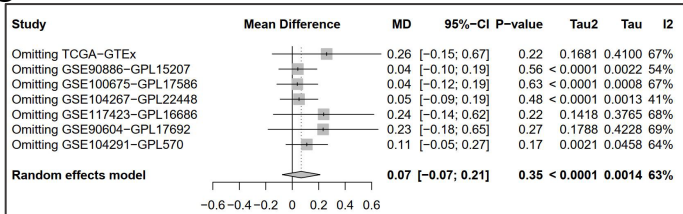**D****FMOD**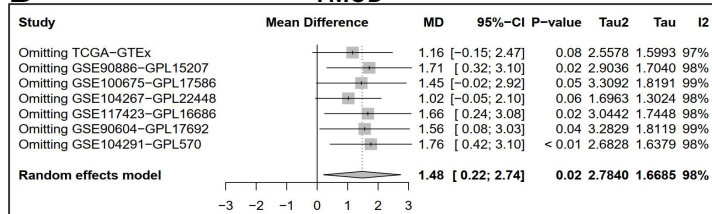**E****IGHG2**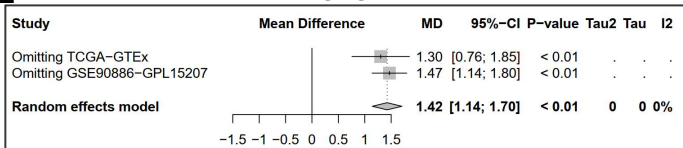

Supplement: Supplementary file 7 — Figure S7 [file CNS-31-e70161-s005.pdf]

**A**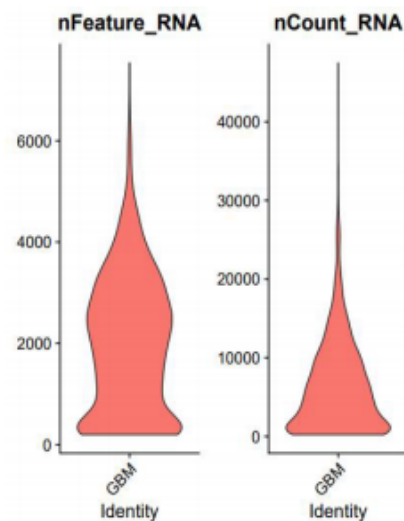**B**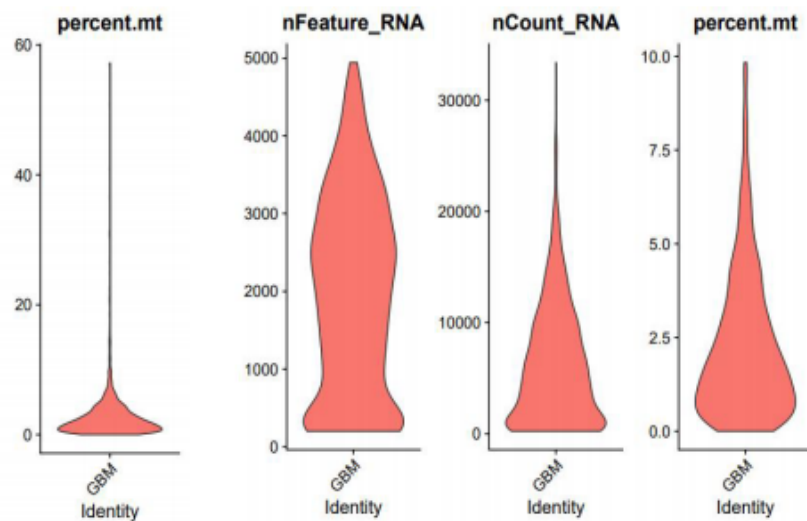**C**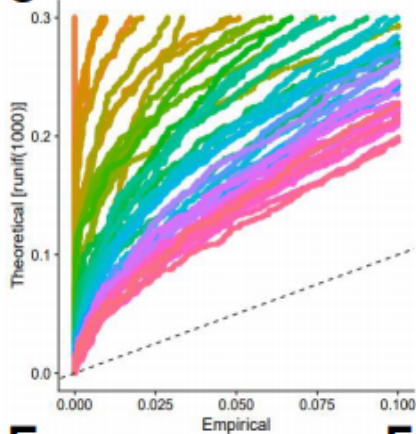**D**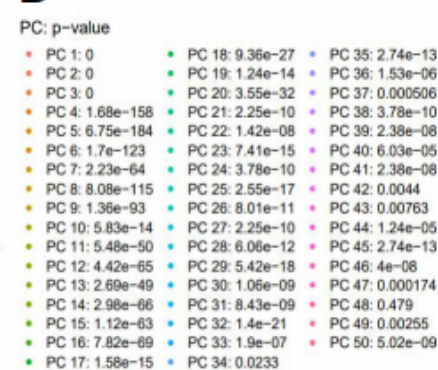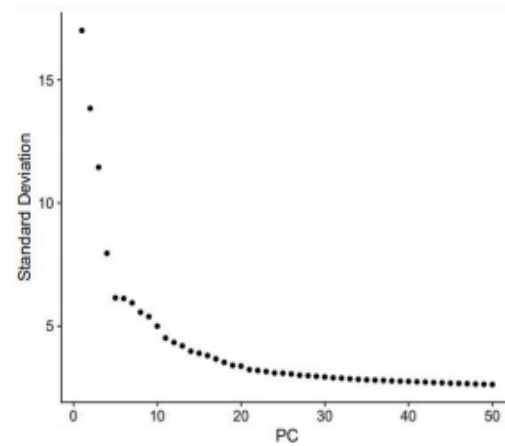**E**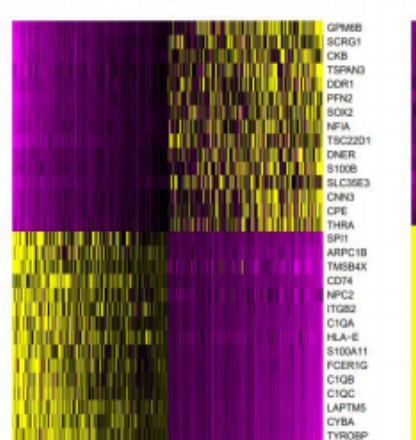**F**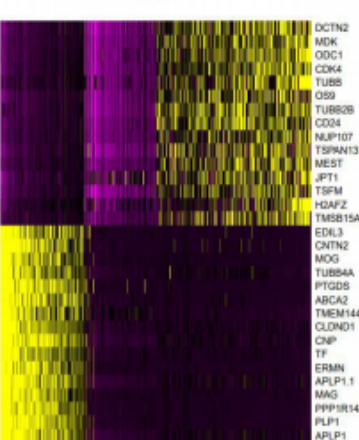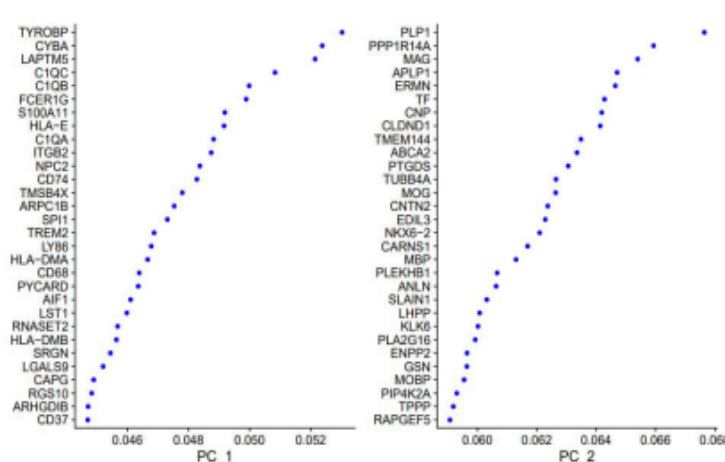**G**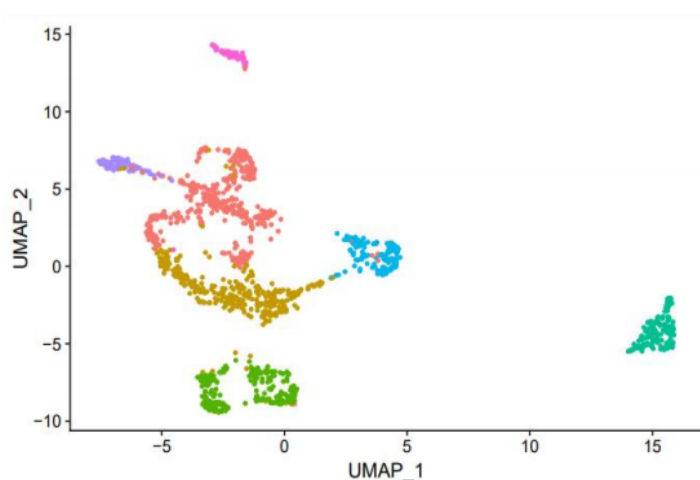**H**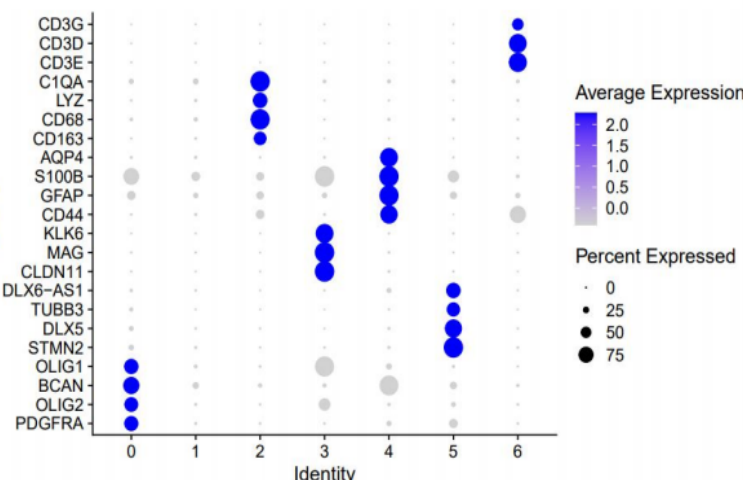

Supplement: Supplementary file 9 — Figure S9 [file CNS-31-e70161-s011.pdf]

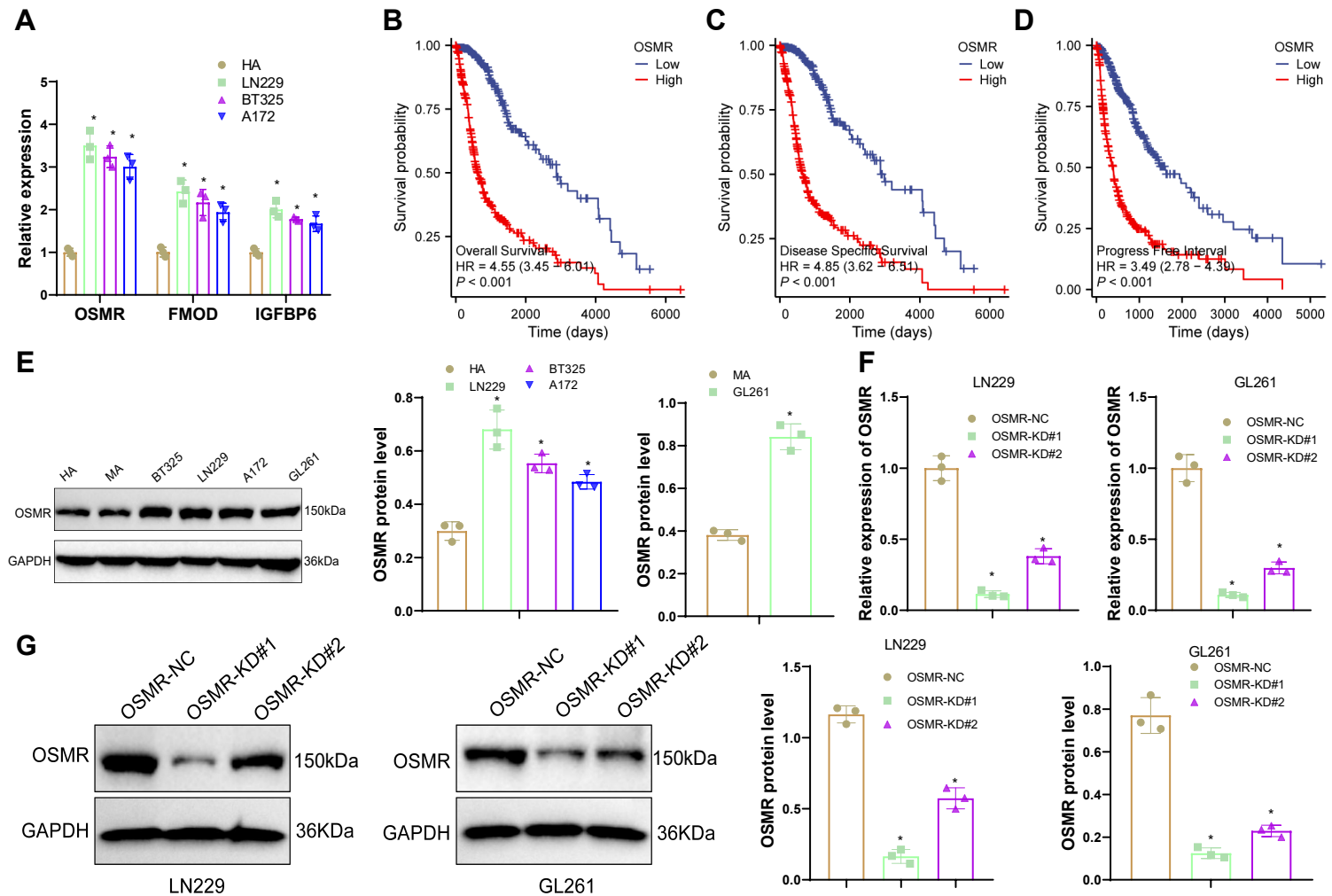

Supplement: Supplementary file 10 — Figure S10 [file CNS-31-e70161-s002.pdf]

**A**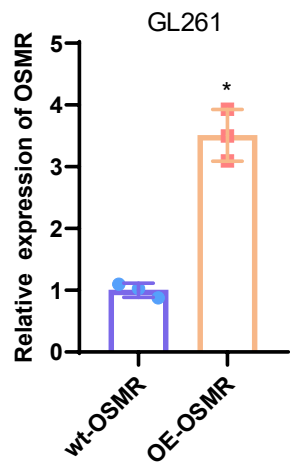**B**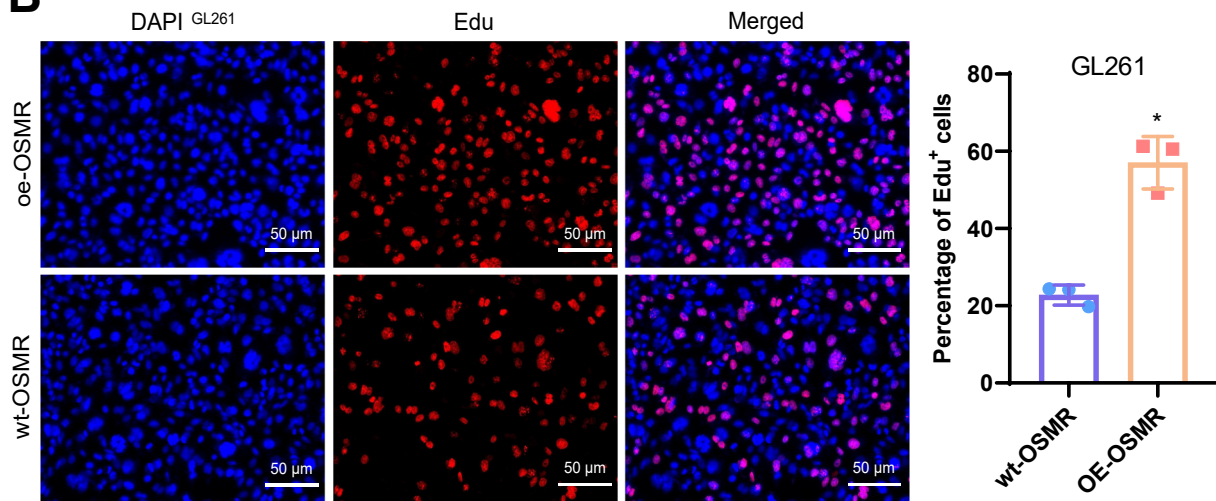**C**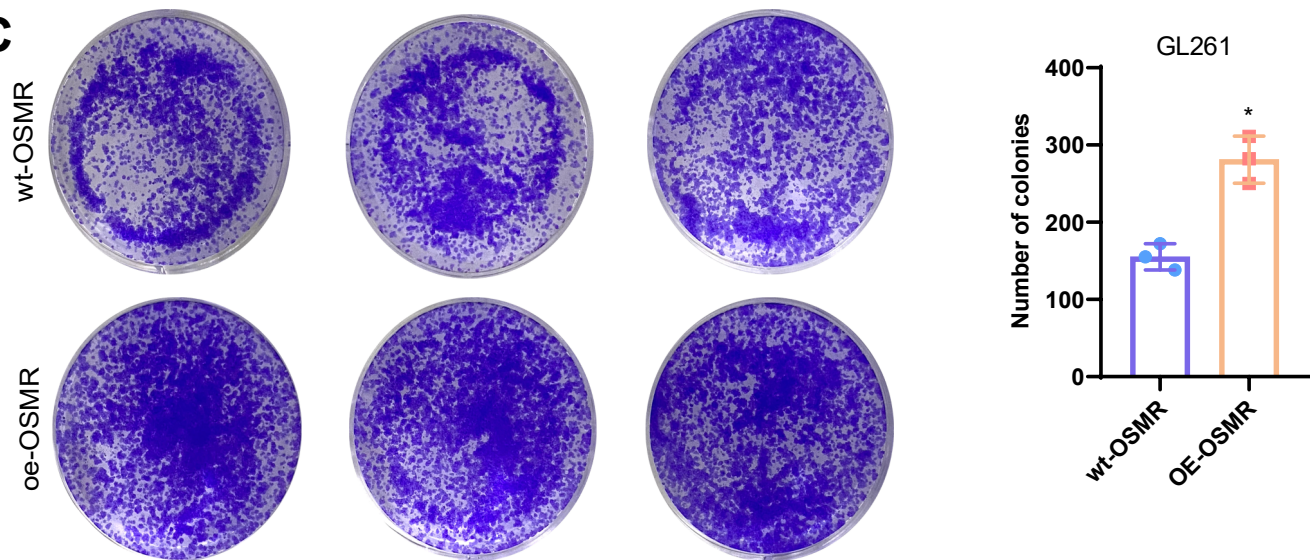

Supplement: Supplementary file 11 — Figure S11 [file CNS-31-e70161-s006.pdf]

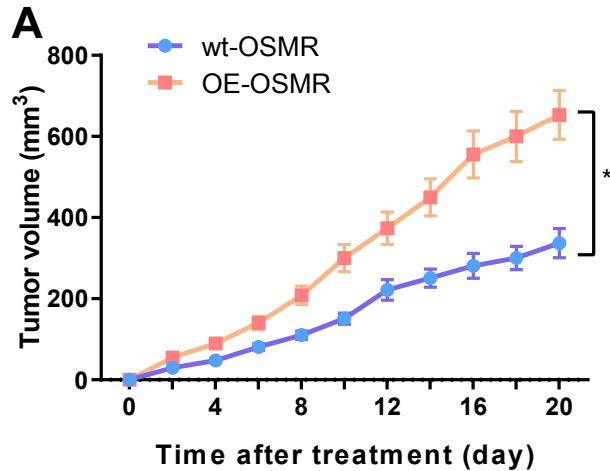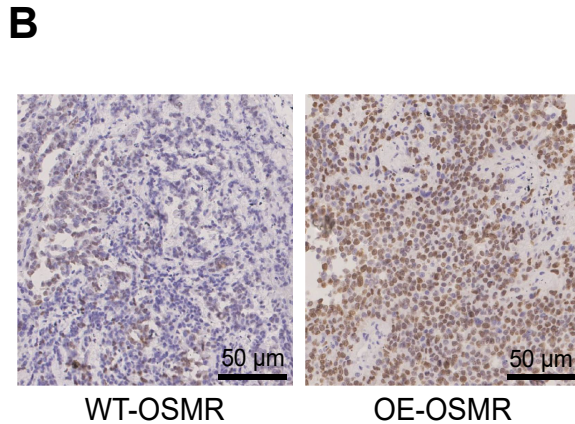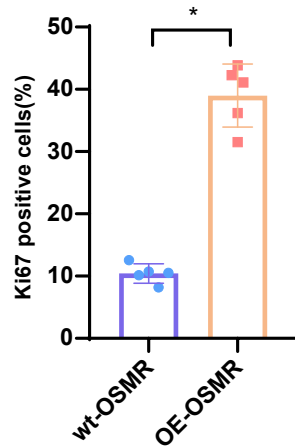

Supplement: Supplementary file 12 — Figure S12 [file CNS-31-e70161-s001.pdf]
