# Supplementary material for: A Risk Model Based on Ferroptosis‐Related Genes OSMR, G0S2, IGFBP6, IGHG2, and FMOD Predicts Prognosis in Glioblastoma Multiforme
Source: CNS Neurosci Ther. 2025 Jan 15;31(1):e70161. doi: 10.1111/cns.70161 (PMC11735466; doi:10.1111/cns.70161)
Supplement: Supplementary file 8 — Figure S8 [file CNS-31-e70161-s003.pdf]

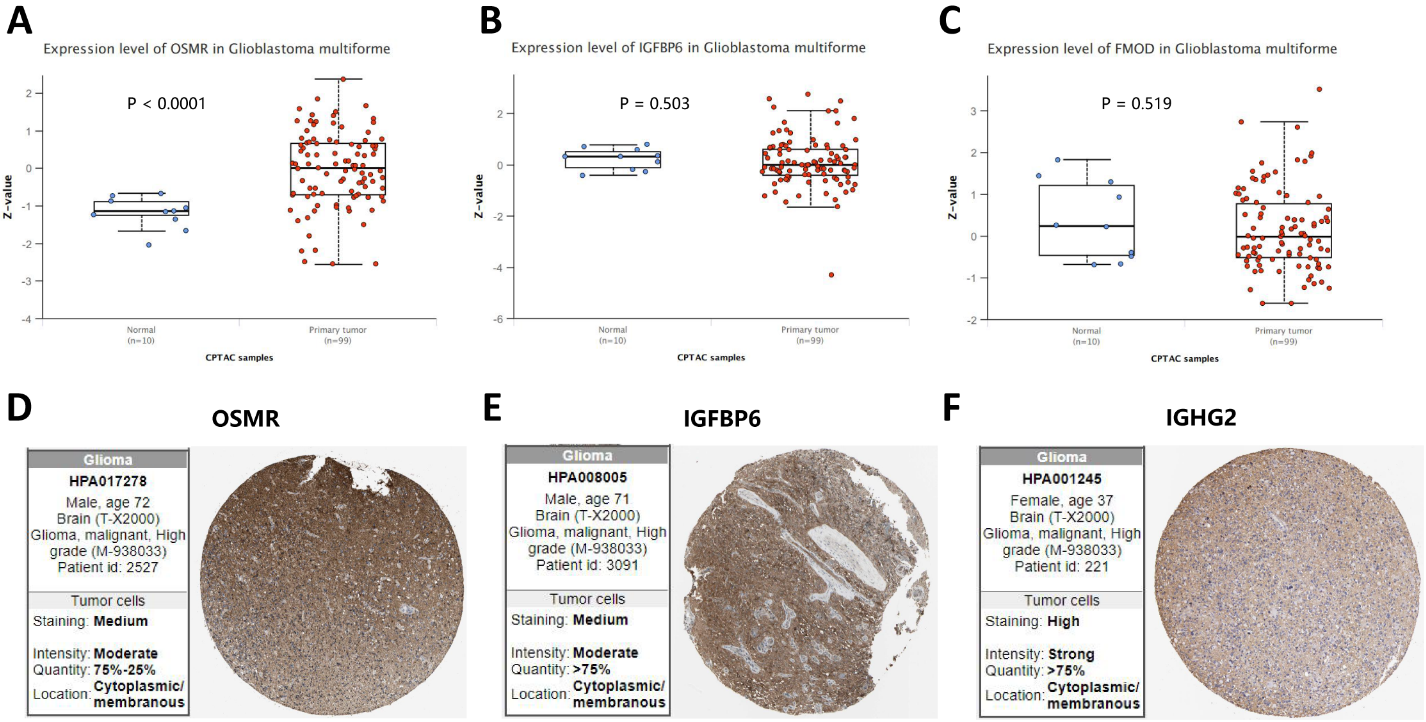
A
Expression level of OSMR in Glioblastoma multiforme
P < 0.0001
Z-value
Normal (n=10)
Primary tumor (n=99)
CPTAC samples
B
Expression level of IGFBP6 in Glioblastoma multiforme
P = 0.503
C
Expression level of FMOD in Glioblastoma multiforme
P = 0.519
D
OSMR
Glioma
HPA017278
Male, age 72
Brain (T-X2000)
Glioma, malignant, High grade (M-938033)
Patient id: 2527
Tumor cells
Staining: Medium
Intensity: Moderate
Quantity: 75%-25%
Location: Cytoplasmic/ membranous
E
IGFBP6
Glioma
HPA008005
Male, age 71
Brain (T-X2000)
Glioma, malignant, High grade (M-938033)
Patient id: 3091
Tumor cells
Staining: Medium
Intensity: Moderate
Quantity: >75%
Location: Cytoplasmic/ membranous
F
IGHG2
Glioma
HPA001245
Female, age 37
Brain (T-X2000)
Glioma, malignant, High grade (M-938033)
Patient id: 221
Tumor cells
Staining: High
Intensity: Strong
Quantity: >75%
Location: Cytoplasmic/ membranous
