# Supplementary material for: A Risk Model Based on Ferroptosis‐Related Genes OSMR, G0S2, IGFBP6, IGHG2, and FMOD Predicts Prognosis in Glioblastoma Multiforme
Source: CNS Neurosci Ther. 2025 Jan 15;31(1):e70161. doi: 10.1111/cns.70161 (PMC11735466; doi:10.1111/cns.70161)
Supplement: Supplementary file 13 — Table S1 Table S2 Table S3 [file CNS-31-e70161-s007.docx]

**Table S1. Sample information of GEO dataset for meta-analysis.**

| **Dataset** | **Platform** | **Normal sample** | **Tumor sample** |
| --- | --- | --- | --- |
| GSE90886 | GPL15207 | 9 | 9 |
| GSE100675 | GPL17586 | 6 | 3 |
| GSE104267 | GPL22448 | 4 | 8 |
| GSE117423 | GPL16686 | 3 | 3 |
| GSE90604 | GPL17692 | 7 | 15 |
| GSE104291 | GPL570 | 2 | 4 |

**Table S2. Lentiviral transfection primer sequences.**

| **Title** | **Sequence** |
| --- | --- |
| OSMR-KD-1(mouse) | 5'-GCATTGATTGTGGACAACCTA-3' |
| OSMR-KD-2(mouse) | 5'-CGTCCGTTACAACTTCAGAAT-3' |
| OSMR-NC(mouse) | 5'-AAGTGGTCAACATGAACTAGG-3' |
| OSMR-KD-1(human) | 5'-GCATTGATTGTGGACAACCTA-3' |
| OSMR-KD-2(human) | 5'-CGAGTTGACTAAGCCTAACTA-3' |
| OSMR-NC(human) | 5'-AGTGTCACATGTACGTAGGAT-3' |

**Table S3. RT-qPCR primer sequence.**

| **Gene** | **Primer** |
| --- | --- |
| OSMR（Human） | F 5'-ACTGGAACCTGCCACAGAGT-3' |
|  | R 5'-TCCAAGCTCACAATTCTCCA-3' |
| IGFBP6（Human） | F 5'- CACAGGATGTGAACCGCAGAGA -3' |
|  | R 5'- CACTGAGTCCAGATGTCTACGG -3' |
| FMOD（Human） | F 5'- CAGTCAACACCAACCTGGAGAAC -3' |
|  | R 5'- CAGCTTGGAGAAGTTCACGACG -3' |
| GAPDH（Human） | F 5'-GGCTGTTGTCATACTTCTCATGG-3' |
|  | R 5'-GGAGCGAGATCCCTCCAAAAT-3 |

Note: F, forward; R, reverse.
